# A group of segmented viruses contains genome segments sharing homology with multiple viral taxa

**DOI:** 10.1128/jvi.00332-25

**Published:** 2025-06-04

**Authors:** Huang Huang, Zhongmei Zhang, Xidan Pang, Qing Tang, Xueqiong Xiao, Jiasen Cheng, Yanping Fu, Yang Lin, Tao Chen, Bo Li, Lei Zhang, Daohong Jiang, Jiatao Xie

**Affiliations:** 1 National Key Laboratory of Agricultural Microbiology, Huazhong Agricultural University 47895, Wuhan, Hubei, China; 2 The Provincial Key Lab of Plant Pathology of Hubei Province, College of Plant Science and Technology, Huazhong Agricultural University 467852 , Wuhan, Hubei, China; 3 Hubei Hongshan Laboratory, Wuhan, Hubei, China; 4 Institute of Plant Protection, Sichuan Academy of Agricultural Sciences 117452, Chengdu, Sichuan, China; 5 Xiangyang Academy of Agricultural Sciences, Xiangyang, Hubei, China; Emory University School of Medicine, Atlanta, Georgia, USA

**Keywords:** segmented virus, mycovirus, genome segmentation, rod-shaped virion

## Abstract

The discovery of diverse segmented RNA viruses through metatranscriptomics has enabled researchers to trace their evolutionary trajectories. However, this effort has been hindered by the limited availability of complete genome sequences and the low similarity of novel viral segments. In this study, we characterized Fusarium asiaticum vivivirus 1 (FaVvV1), a +ssRNA mycovirus with 10 monocistronic RNA segments (S1 to S10, encoding VP1 to VP10), present in the phytopathogenic fungus *Fusarium asiaticum*. VP1 and VP2 exhibit homology with replication proteins of martellivirals, while VP3 and VP5 share similarities with the nuclear inclusion protein a and the cylindrical inclusion helicase of potyvirids, respectively. FaVvV1 forms rod-shaped virions, with VP8 functioning as a structural protein resembling the helical capsid of potyvirids and closterovirids. To explore the conservation and evolution of viviviruses, we mined 23 public Sequence Read Archive (SRA) datasets, identifying 29 vivivirus-related viruses (vivivirids) comprising 186 viral segments. VP1 (methyltransferase and RdRP domain), VP2 (methyltransferase and superfamily 1 helicase domain), VP3 (chymotrypsin-type serine protease domain), VP5 (superfamily 2 helicase domain), and VP8 (helical capsid) were identified as conserved hallmark proteins of viviviruses. Phylogenetic and structural analyses suggest that multiple genome segmentations and gene/domain duplications were involved in the evolution of vivivirids. VP3, VP5, and VP8 might share a common ancestor with potyvirids. These findings highlight the intricate evolutionary mechanisms underlying segmented virus diversity and adaptation.

**IMPORTANCE:**

Metaviromics has greatly expanded our understanding of viral diversity, including segmented or multipartite RNA viruses with genomes composed of multiple segments. However, virome analyses often fail to detect genomic segments beyond the RdRP, likely due to their low similarity to known viruses. We characterized a group of segmented, potentially multipartite, +ssRNA viruses, with Fusarium asiaticum vivivirus 1 as a representative; most of these viruses likely infect fungi. Through structural and evolutionary analysis of the five core segments of viviviruses, our findings highlight key aspects of vivivirus evolution, including genome segmentation, gene and domain duplications, and segments with multiple evolutionary origins.

## INTRODUCTION

Mycoviruses are viruses that replicate in fungi and infect all major taxonomic groups of fungi (1). Most mycoviruses remain cryptic or latent in their hosts, but some influence host phenotypes to varying degrees (2, 3). Hypovirulence-associated mycoviruses have attracted significant interest as potential biocontrol agents, with research focusing on various phytopathogenic fungi, including *Cryphonectria parasitica*, the causative agent of chestnut blight (4, 5). Mycovirus research also expands our understanding of viral diversity and evolution (6). One notable example is the discovery of ambiviruses, whose circular RNA genome contains active ribozymes and encodes an RNA-dependent RNA polymerase (RdRP) protein, representing a unique hybrid between viroid-like RNAs and RNA viruses (7). Additionally, fungi harbor diverse segmented +ssRNA viruses. For example, splipalmiviruses exhibit unique segmentation of the palm subdomain of the RdRP protein (8, 9), while polymycoviruses (10, 11) and hadakaviruses (12), despite their enigmatic evolutionary origins, share a common ancestor with animal caliciviruses. The bi-segmented ormycoviruses contain a novel RdRP domain that cannot be classified into any established RNA virus phyla (13).

A novel group of segmented +ssRNA viruses within *Martellivirales*, termed vivivirus, was recently identified using next-generation sequencing (NGS) techniques for viral discovery. Initially, four viviviruses, Plasmopara viticola lesion-associated vivivirus 1–4, were reported as bi-segmented viruses. One segment encodes a protein containing a putative methyltransferase (Mtr) domain and an RdRP domain, while the other encodes a protein with a conserved Mtr domain and a superfamily 1 helicase (SF1H) domain (14). These segments showed similarities to their counterparts in *Virgaviridae*, a family of plant +ssRNA viruses characterized by the Mtr-SF1H-RdRP replication module, leading to the naming of this virus group as vivivirus (14). Subsequently, two related viviviruses, Aspergillus flavus vivivirus 1 (AfVvV1) and Aspergillus fumigatus RNA virus 1 (AfRV1), were identified as tri-segmented viruses with one segment encoding a hypothetical protein (8, 15). Furthermore, over 40 vivivirus-like segments identified from fungi-, plant-, and insect-associated viromes have been published or submitted to the GenBank database, as summarized in Table S1. Most of these viral segments encode either (i) proteins containing Mtr-RdRP or Mtr-SF1H domains or (ii) hypothetical proteins (16–22). An exception is wheat-associated vipovirus, which was reported to possess two genomic segments: RNA1 encoding an Mtr-RdRP domain similar to that of *Virgaviridae* and RNA2 containing a DEAD-like helicase domain (16). Furthermore, the helicase domain in RNA2 resembles the cylindrical inclusion (CI) helicase of potyviruses, which belongs to the superfamily 2 helicase (SF2H) group (16, 23). These findings suggest an oversight regarding the multi-segmental characteristics of these viruses and that fungi may serve as primary hosts for viviviruses. In 2023, the genome of AfVvV1 was found to consist of 12 segments (24), providing a more complete picture of its genomic complexity. Nevertheless, the biological properties, virions, and evolutionary origins of viviviruses remain elusive.

Fusarium head blight (FHB), caused by *Fusarium* species, is an important cereal disease worldwide, resulting in serious yield and quality losses. Additionally, it causes severe mycotoxin contamination of wheat, posing a great threat to food safety and the health of both humans and livestock. Within the *Fusarium graminearum* species complex causing FHB, *Fusarium asiaticum* and *Fusarium graminearum* exhibit distinct geographic distributions. *F. asiaticum* is predominantly found in rice-growing regions of Asia, while *F. graminearum* is more commonly associated with corn-growing areas worldwide (25–28). Due to differences in geographical distribution, most studies on mycoviruses infecting *Fusarium* species have focused on *F. graminearum* (29), identifying five hypovirulence-associated mycoviruses. These include two betachrysovirus strains (30, 31), one hypovirus (32), one fusarivirus (33), and one gemytripvirus (34). Recently, two viromic studies of FHB-associated pathogens identified 34 mycoviruses, including novel viruses in *Yueviridae* and *Phenuiviridae*. Moreover, the majority of these viruses were found to infect *F. asiaticum* and *F. culmorum* (35, 36).

In this study, we identified and characterized Fusarium asiaticum vivivirus 1 (FaVvV1), a 10-segmented +ssRNA virus with rigid rod-shaped virions, isolated from the hypovirulent *F. asiaticum* strain BZ6. Biological assays demonstrated that FaVvV1 infection potentially affected the growth rate and virulence of *F. asiaticum*. By analyzing 29 vivivirus-related viruses assembled from SRA data, we aimed to predict functional conservation and elucidate the evolution of vivivirids (including vivivirus and vipovirus). Our findings highlighted the remarkable diversity of viviviruses with more than five segments and provided insights into their evolution.

## RESULTS

### Identification of a novel mycovirus belonging to *Martellivirales*

**Fig 1:**
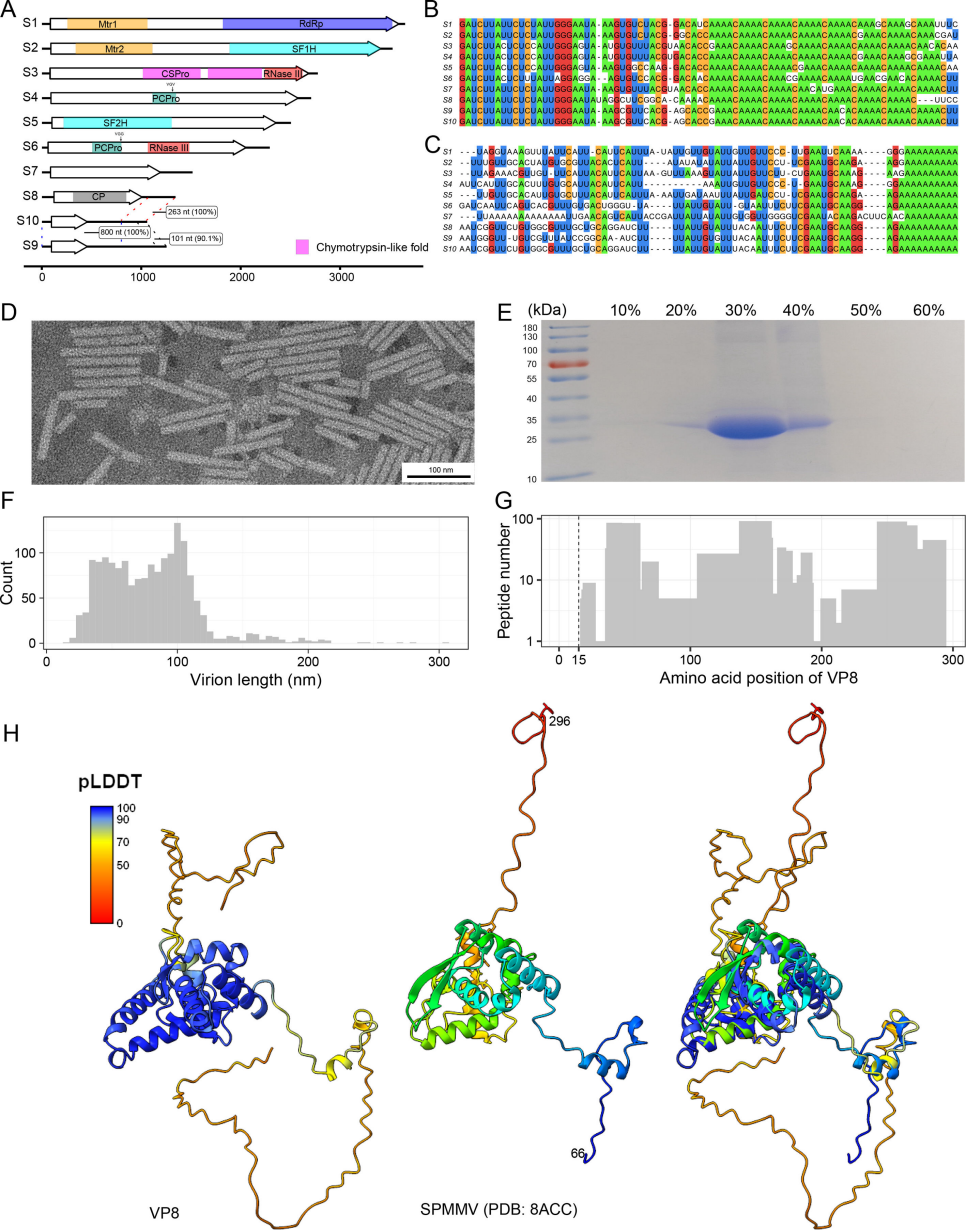
Genome features and virion of FaVvV1. (**A**) The 10 complete genomic segments of FaVvV1. Putative ORFs are presented as white arrows, and the protein domains and annotations are marked using colored rectangles. Regions with colored dashed lines indicate high nucleotide similarity among RNA segments S8, S9, and S10, with length and similarity information shown in boxes. Predicted papain-like cysteine protease cleavage sites in VP4 and VP6 are indicated by arrows. Mtr1, Methyltransferase (Mtr) domain in VP1. Mtr2, Mtr domain in VP2. RdRP, RNA-dependent RNA polymerase. SF1H, superfamily 1 helicase. SF2H, superfamily 2 helicase. CSPro, chymotrypsin-type serine protease. PCPro, papain-like cysteine protease. CP, capsid. The figure was drawn using the R package gggenes 0.5.1 (https://cran.r-project.org/web/packages/gggenes). (**B**) Alignment of the 5′ terminal region of FaVvV1. (**C**) Alignment of the 3′ terminal regions of FaVvV1. Long poly(A) tails at the 3′ termini were trimmed for alignment. Residues with >50% identity are colored. (**D**) Morphology of FaVvV1 virions. (**E**) SDS-PAGE of FaVvV1 structural proteins. (**F**) FaVvV1 virion length distribution. (**G**) Peptide distribution of the predicted capsid ORF. The first peptide match location is marked with a dotted line. (**H**) Structural alignment of the capsids of FaVvV1 and sweet potato mild mottle virus (SPMMV). Left, ColabFold2-predicted structure of FaVvV1 capsid, colored according to the pLDDT score. Center, coat protein of sweet potato mild mottle virus (PDB: 8ACC) in wheat color (37). Right, superposition of the two structures with TM-align.

To explore the diversity of mycoviruses associated with FHB pathogens, 150 fungal strains were isolated from wheat spikes exhibiting typical FHB symptoms. Most strains were identified as *F. asiaticum* (84/150, 56%) or *F. graminearum* (63/150, 42%). Approximately 16 different mycoviruses with partial or complete genomes were identified through virome analysis of 150 *Fusarium* strains (unpublished data). Among contigs of these viruses, seven showed similarities to those of AfRV1 and AfVvV1. We confirmed the presence of these contigs in strain BZ6 through RT-PCR, suggesting that they may constitute a single viral genome, which was temporarily named Fusarium asiaticum vivivirus 1 (FaVvV1). Considering the multi-segmented genome characteristics of the reported viviviruses, the total RNA of strain BZ6 was sequenced using a metatranscriptomic approach to identify additional viral genome segments. We scrutinized and annotated all assembled contigs, with particular attention to orphan or dark sequences. These dark contigs were further validated using RT-PCR to determine if they were segments of FaVvV1. Finally, 10 contigs were confirmed as part of the FaVvV1 genome, as they were detected in strain BZ6 and shared a conserved 5′ terminal sequence. The complete FaVvV1 genome, comprising segments S1 to S10 with lengths ranging from 1,060 to 3,654 nucleotides (nt), was obtained using RACE methods (Fig. 1A). All genomic segments of FaVvV1 shared a conserved motif of 80 nt at the 5′ end and a poly(A) tail at the 3′ end (Fig. 1B and C). The conserved 5′ terminal sequence includes a (CAAAA)_7–8_ repeat, also observed in AfVvV1 (24). Unexpectedly, the first 800 nt at the 5′ end of S9 and S10 were identical, the last 101 nt at the 3′ end of S9 and S10 shared 90% identity, and the last 263 nt at the 3′ end of S8 and S10 were identical (Fig. 1A). The sequences of S8, S9, and S10 were validated by Sanger sequencing to rule out assembly errors. These results suggest that genome recombination and duplication events occurred during the evolution of S9 and S10. The predicted ORFs of S9 and S10 were identical and encoded a hypothetical protein of 120 amino acids. A comprehensive functional analysis of the viral proteins encoded by S1–S8 is provided in the following sections.

### FaVvV1 virions display a rigid rod-shaped morphology

Although numerous viviviruses have been reported, whether they form virions and their potential virion morphology remain unconfirmed. To date, only one study has attempted to purify AfVvV1 virions from a strain co-infected with Aspergillus flavus deltaflexivirus (15). The researchers concluded that AfVvV1 might produce isometric virions, whereas Aspergillus flavus deltaflexivirus virions were probably flexuous filaments (15). We purified the FaVvV1 virions from strain BZ6 by sucrose gradient centrifugation. Under transmission electron microscopy, rod-shaped virions with a width of 12–15 nm and a length of 30–110 nm were observed (Fig. 1D and F). All 10 FaVvV1 segments were detected by RT-PCR using purified virion RNA as the template (Fig. S1A). However, the purified virion RNA exhibited a smeared electrophoretic pattern, suggesting degradation by RNases or mechanical fragmentation during isolation. SDS-PAGE analysis of purified virions showed a distinct band of ~30 kDa in the 30% sucrose gradient fraction (Fig. 1E). Mass spectrometry identified this band as VP8, with all predicted VP8 regions covered by peptides except for the N-terminal 15-aa region (Fig. 1G). The absence of N-terminal peptide coverage and the presence of an N-acetylated peptide at the 13th position imply that the capsid may utilize a second AUG of the predicted ORF as the translation start site (Fig. S1B).

To further investigate the relationship between VP8 and other coat proteins (CPs) of rod-shaped filamentous virions, we searched for remote VP8 homologs in the UniProt-SwissProt-vir70 and PDB30 databases using the HHpred server, as BLASTP yielded no functional information. The results suggested that VP8 shares 10%–20% identity with CPs of closterovirids and potyvirids, which typically form flexuous filamentous particles (Table S2). Furthermore, the predicted 3D structure of the FaVvV1 CP was also similar to those of sweet potato mild mottle virus (SPMMV), sweet potato feathery mottle virus, and potato virus Y in the family *Potyviridae* based on Foldseek search results (Table S3). The predicted CP monomer structure displayed a conserved globular domain with seven α-helices and one β-hairpin in the central region, and disordered N- and C-terminal regions (Fig. 1H; Fig. S1C), consistent with previously reported structures of helical capsids (37–39). Although the CPs of FaVvV1 and SPMMV exhibited low sequence identity, structural alignment showed a root mean square deviation (RMSD) of 4.07 Å and a template modeling score (TM-score) of 0.61, indicating a similar fold. However, pairwise sequence and structural alignment showed that the FaVvV1 CP forms a distinct cluster, separate from other virus families such as *Potyviridae*, *Alphaflexiviridae*, and *Closteroviridae* (Fig. S2). In summary, our findings demonstrate that FaVvV1 forms rod-shaped virions with a unique capsid protein.

### Vivivirus-related viruses and novel-segmented viruses were discovered in public databases

**Fig 2:**
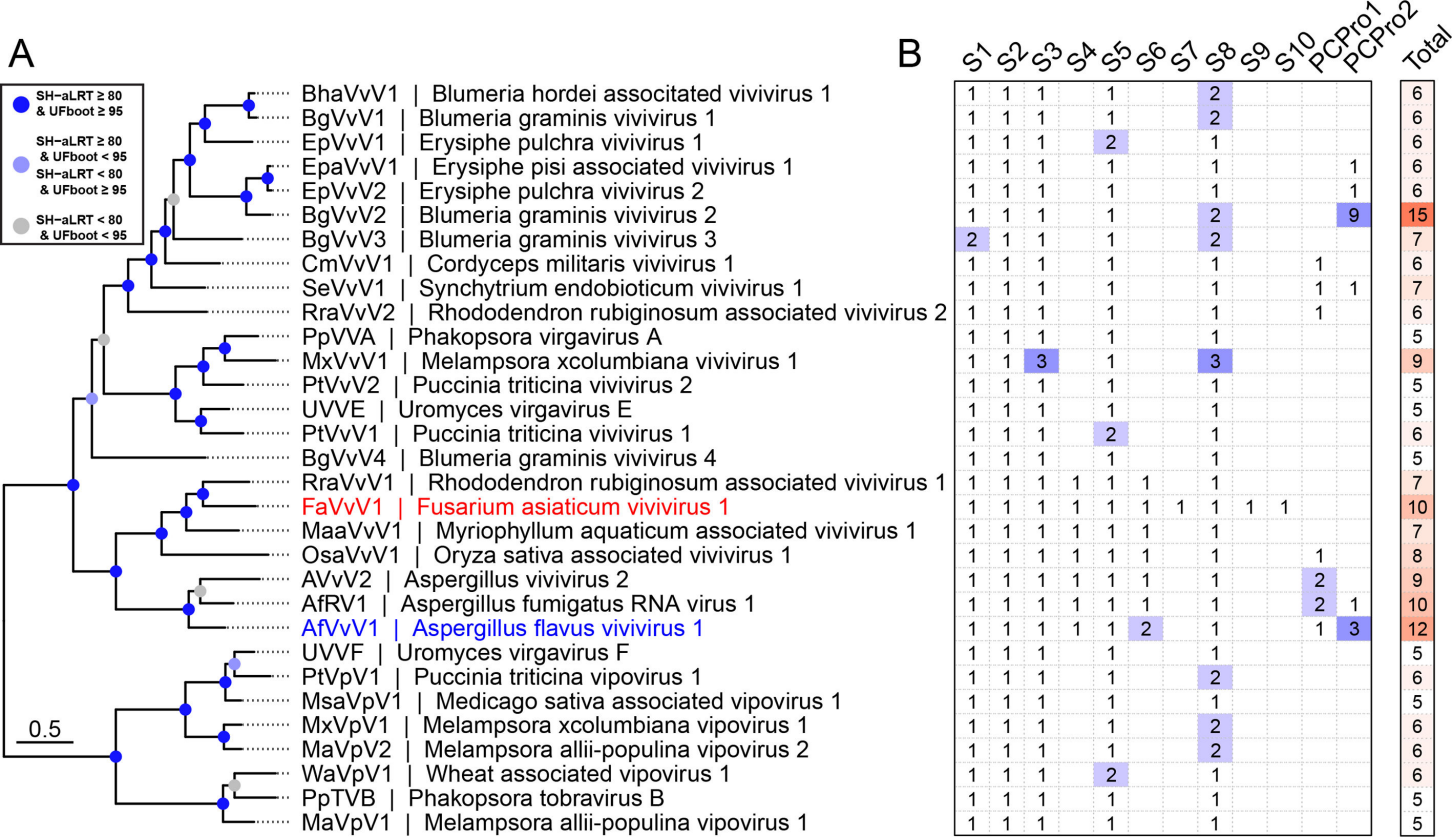
Newly discovered viviviruses and vipoviruses. (**A**) Phylogenetic analysis of assembled viruses based on the Mtr1-RdRP domain. FaVvV1 described here is highlighted in red. Another recently reported vivivirus, AfVvV1, with a complete genome is in blue. The best-fit model is Q.pfam + F + I + G4 according to the Bayesian information criterion. For better clarity, nodes are color-coded based on their branch support values: nodes with SH-aLRT ≥80 and UFboot ≥95 are marked in blue, those with either SH-aLRT ≥80 and UFboot <95 or SH-aLRT <80 and UFboot ≥95 are shown in light blue, and branches where both SH-aLRT <80 and UFboot <95 are displayed in gray. (**B**) Genome contents and RNA segment numbers of assembled viviviruses and vipoviruses with FaVvV1 as a reference. PCPro1 and PCPro2 refer to homologous RNA segments containing a papain-like cysteine protease, excluding PCPro-containing segments S4 and S6.

Since most reported vivivirus-related virus genomes are incomplete, obtaining detailed information on the gene function and evolution of FaVvV1 has been challenging. Therefore, we utilized the recently published Serratus to identify novel vivivirus-related viruses from public SRA data (40). Finally, we assembled 29 segmented vivivirus-related viruses, including 21 viviviruses and 8 vipoviruses, from 23 SRA datasets of plants or fungi (Table S4). Based on phylogenetic analysis of VP1, the vivivirus-related viruses formed two clades, vivivirus and vipovirus (Fig. 2A). VP1, VP2, VP3, VP5, and VP8, encoded by FaVvV1 segments S1, S2, S3, S5, and S8, were conserved across viviviruses and vipoviruses (Fig. 2; Table S4). In addition, duplicated segments, which refer to segments that are homologous or share the same domain in the same virus, were frequently found in the assembled viviviruses and vipoviruses (14/31). Most duplication events (8/18) involved capsid-encoding segments similar to duplication of capsids in the genome of closterovirids. A notable example was the duplication events of S3 and S8 found in Melampsora × columbiana vipovirus 1 and Melampsora × columbiana vivivirus 1, both infecting the same strain of *Melampsora* × *columbiana*. As *M*. × *columbiana* is a natural hybrid of two *Melampsora* spp. (41), segment reassortment may have contributed to the observed duplication events (Fig. 2B).

We identified six novel segmented viruses in the SRA dataset from Pucciniomycetes fungi, with genomic segments exhibiting limited similarity to VP1, VP2, and CP of FaVvV1 (Table S5). These viruses were provisionally named “puccinivirus.” Despite the different genomic segment compositions among viviviruses, vipoviruses, and pucciniviruses, the separation of the RdRP domain from the Mtr-SF1H domain was conserved across all three groups, implying that at least a similar genome segmentation event was involved (Table S4 and S5; Fig. S3A). Except for S1 and S2, pucciniviruses showed conservation in certain segments, which encode an unknown helicase-like domain, an RNase III domain, or a capsid resembling those of alphaflexivirids or closterovirids (Table S5; Fig. S3A and B). Other RNA segments of pucciniviruses were conserved only within the subclade of puccinivirus, and their encoded proteins lacked identifiable domains (Table S5).

### A second methyltransferase domain in viviviruses, vipoviruses, jiviviruses, and pucciniviruses

**Fig 3:**
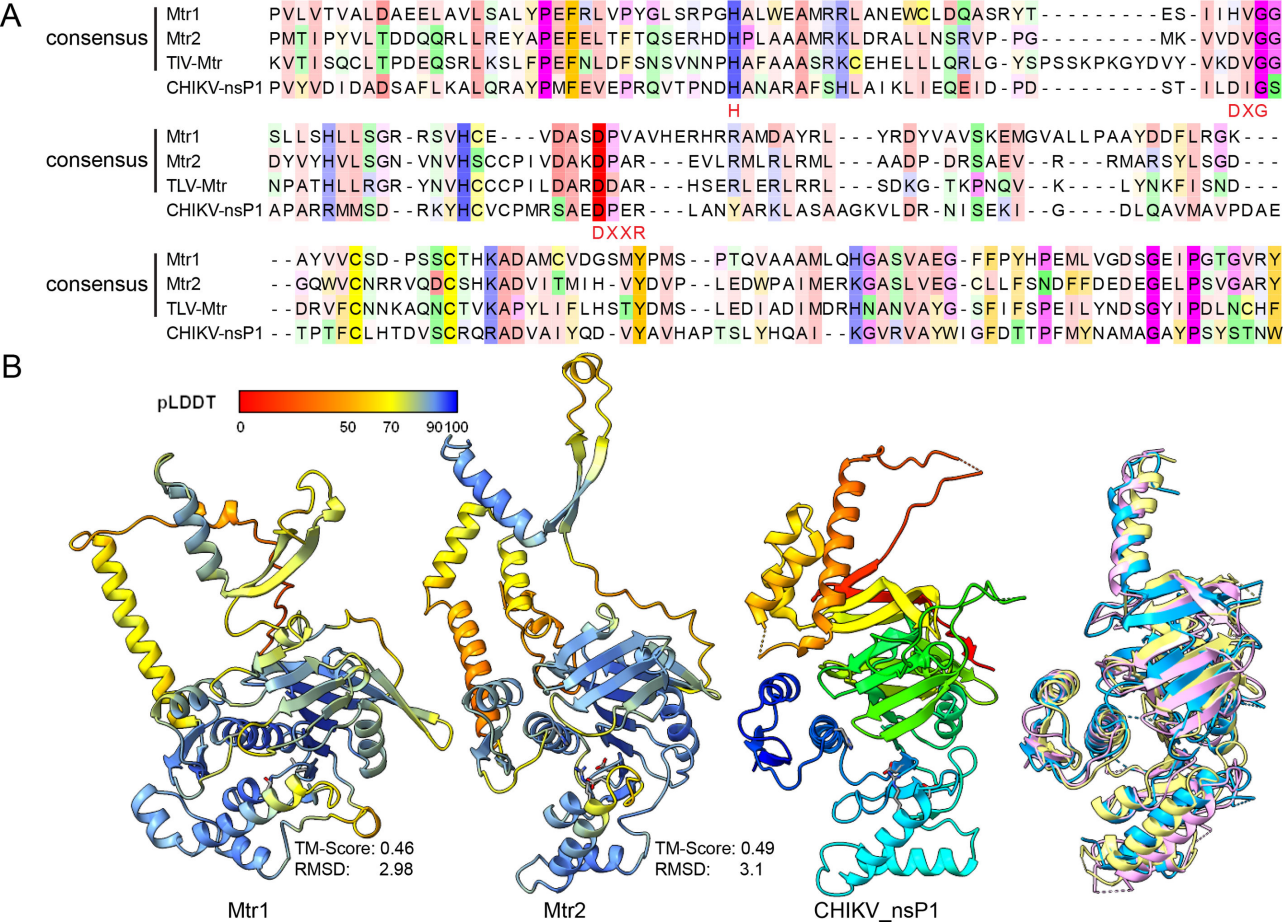
Comparison of Mtr1 and Mtr2 domains. (**A**) Alignment of Mtr domains in S1 and S2 of vivivirus. Only the consensus and core sequences of the Mtr domain are shown here due to space limitations. Motifs involved in capping activity are highlighted in red. (**B**) The 3D structures of the Mtr domains of FaVvV1 are aligned to the known viral methyltransferase nsP1 of Chikungunya virus. Structural similarity of Mtr1 and Mtr2 compared to CHIKV_nsP1 is noted as TM-score (template modeling score) and RMSD (root mean square deviation), as calculated using jFATCAT-flexible (42). The structures of Mtr1 and Mtr2 are colored according to the value of predicted local distance difference test (pLDDT), and the structure of CHIKV_nsP1 (PDB: 7 X 01_A) is colored using a rainbow scheme (N-terminus in blue).

Viviviruses were previously predicted to contain two putative methyltransferase domains: Mtr1 in the N-terminus of VP1 and a canonical Mtr domain (Mtr2) in the N-terminus of VP2, as suggested in AfRV1 (8). To explore the relationship between Mtr1 and Mtr2, we compared these Mtr domains of viviviruses. Mtr2 was homologous to the Mtr domain in *Martellivirales* and contained conserved motifs for cap transfer (H) and S-adenosylmethionine binding (DXG, DXXR) (Fig. 3A). However, Mtr1 and Mtr2 shared low identity during HMM-HMM alignment using the hhalign tool, with 11% identity between Mtr1 and Mtr2 in FaVvV1 and 20% identity between consensus sequences of Mtr1 and Mtr2 of viviviruses (Fig. S4). The predicted 3D structures of the Mtr domains of S1 and S2 were similar to that of non-structural protein 1 (nsP1) of Chikungunya virus, based on Foldseek search results, the only available viral methyltransferase structure in *Martellivirales* (Fig. 3B). However, unlike Mtr2, Mtr1 of viviviruses and vipoviruses generally lacked the conserved motifs (H, DXG, DXXR) (Fig. S4 and S5). These results indicated that Mtr1 shows limited similarity to Mtr2 in sequence and structure, suggesting it is a putative novel methyltransferase.

Given that both the newly identified pucciniviruses and segmented fungal or plant-associated jiviviruses (43, 44) exhibit a genome arrangement where the RdRP domain is separated from the Mtr-SF1H domain, we speculated that these viruses may also have evolved an Mtr domain that is not readily detectable. Alignment of N-terminal regions upstream of the RdRP domain in segment S2 (Mtr1-RdRP) of pucciniviruses and jiviviruses revealed sequence conservation within each group (Fig. S5). However, the motifs in Mtr1 of pucciniviruses and jiviviruses differed from those in martellivirals despite an HHpred search revealing similarity to the Mtr domain of martellivirals (Fig. S5 and S6). Furthermore, four pucciniviruses had an RNA segment encoding a protein with a single Mtr domain, similar to the Mtr1 in the Mtr1-RdRP domain, possibly resulting from a truncation or segmentation event (Table S5; Fig. S5). These results suggest that a second methyltransferase domain (Mtr1), distinct from the canonical Mtr domain in martellivirals, has evolved extensively in RdRP-containing proteins of segmented +ssRNA viruses (viviviruses, vipoviruses, jiviviruses, and pucciniviruses) through gene duplication-diversification or other unknown mechanisms.

### Vivivirus and vipovirus cluster within *Martellivirales* based on phylogenetic analysis of RdRP, Mtr, and SF1H domains

**Fig 4:**
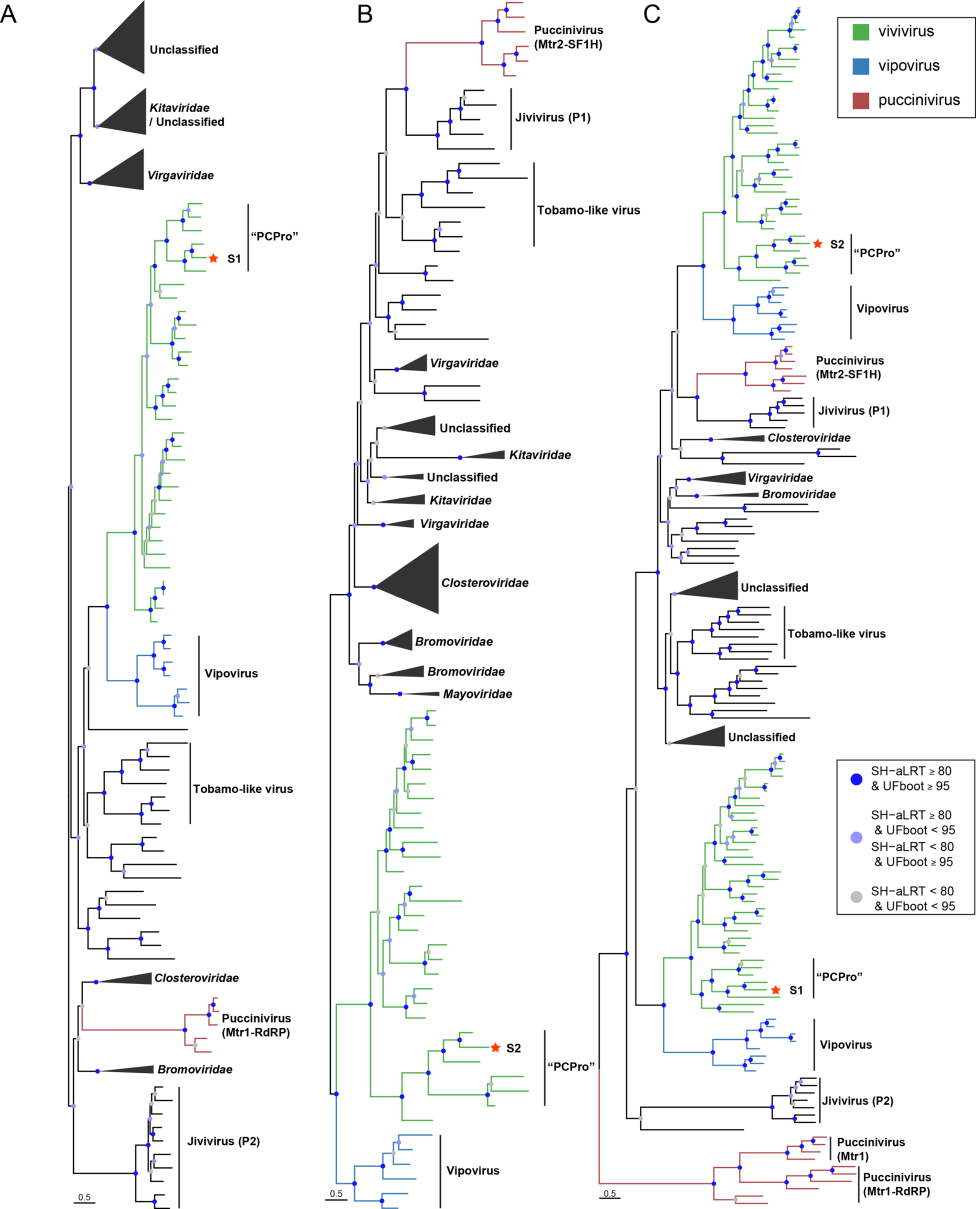
Phylogenetic analysis of RdRP (**A**), SF1H (**B**), and Mtr (**C**) domains encoded by S1 and S2 of vivivirids and pucciniviruses. All trees are midpoint-rooted. S1 and S2 of FaVvV1 are highlighted with a red five-pointed star. The “PCPro” subclade refers to a group of vivivirus-shared segments encoding papain-like cysteine protease. The best-fit models of RdRP, SF1H, and Mtr were Q.pfam + F + R9, Q.pfam + F + R8, and Q.pfam + F + R8, respectively, according to Bayesian information criterion. The complete tree is presented in .

To grasp the evolutionary relationships between viviviruses and martellivirals, phylogenetic analyses were conducted based on three conserved domains: RdRP, Mtr, and SF1H. The RdRP domains of viviviruses and vipoviruses formed two closely related clades alongside the “tobamo-like viruses” (Fig. 4A; Fig. S7A). However, caution should be exercised when interpreting the relationship between vivivirids and “tobamo-like viruses” because the branching position between the two groups has low SH-aLRT (<80) and UFBoot support values (<95) (Fig. 4A; Fig. S7A). The SF1H domains of viviviruses and vipoviruses formed a distinct clade that was adjacent to *Bromoviridae* and *Mayoviridae* in the midpoint-rooted tree (Fig. 4B; Fig. S7B). The Mtr2 domains in VP2 (S2) of viviviruses and vipoviruses formed a distinct clade within *Martellivirales*, whereas the Mtr1 domain in VP1 (S1) grouped into a separate lineage (Fig. 4C; Fig. S7C). Given the close evolutionary relationship between viviviruses and vipoviruses, we propose a new family, Viviviridae, comprising two genera: Vivivirus and Vipovirus.

The RdRP domains of pucciniviruses appear more similar to those of jiviviruses than to those of vivivirids (i.e., viviviruses and vipoviruses in the proposed Viviviridae) in *Martellivirales* although the phylogenetic position of pucciniviruses remains ambiguous (Fig. 4A). The Mtr2 and SF1H domains in VP1 of pucciniviruses clustered with their homologous domains in P1 of jiviviruses (Fig. 4B and C). The Mtr1 domains encoded by the S2 segment and the truncated segment of the four pucciniviruses formed a single clade, further supporting the hypothesis that the truncated segments evolved from the S2 segment in pucciniviruses (Fig. 4C). Similar to the Mtr1 domains encoded by S1 of vivivirids, the Mtr1 domains of pucciniviruses and jiviviruses (P2) occupy distinct evolutionary positions compared to the canonical viral methyltransferases in *Martellivirales* (Fig. 4C). These results suggest that pucciniviruses may be distantly related to jiviviruses, which is further supported by the observation that the segment containing the Mtr2-SF1H domain in both pucciniviruses and jiviviruses is longer than the segment containing the Mtr1-RdRP domain (Fig. S3A). However, whether vivivirids, pucciniviruses, and jiviviruses share a common unsegmented progenitor virus in *Martellivirales* remains unknown.

### VP3 and VP5 are homologous to their counterparts in potyvirids

**Fig 5:**
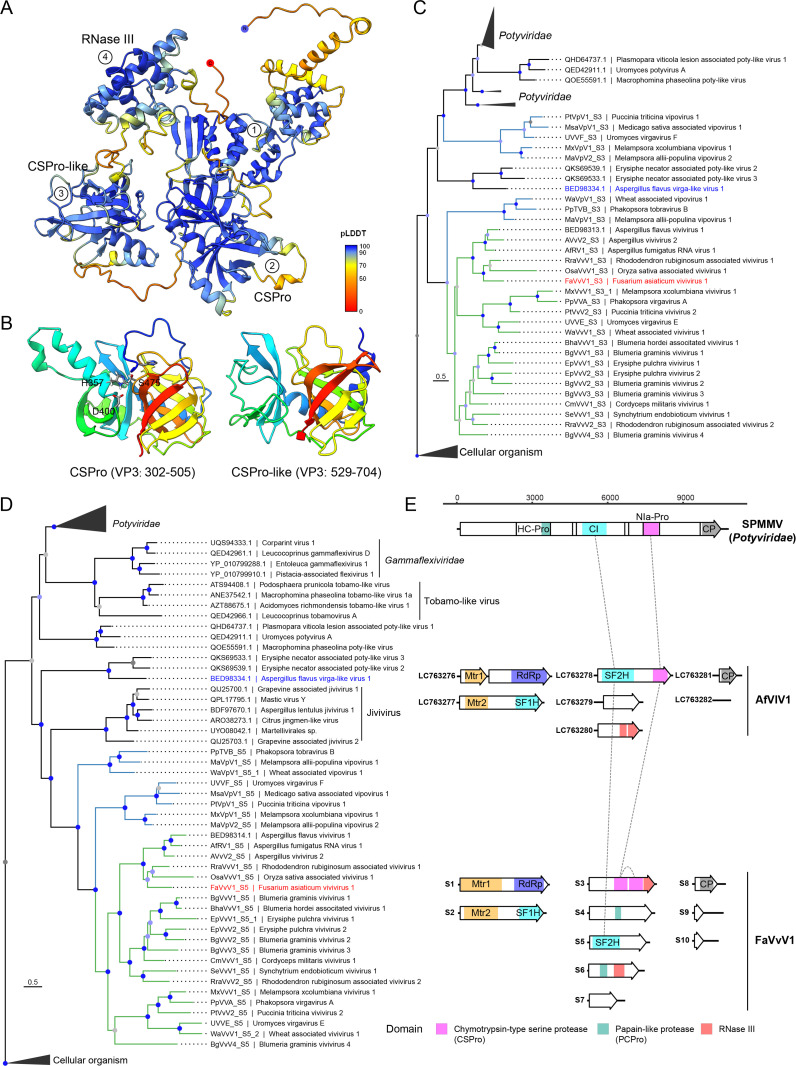
CSPro of VP3 and SF2H of VP5 share homology with potyvirid protein. (**A**) AlphaFold2-predicted 3D structure of VP3. The structure is colored according to the pLDDT values. CSPro, chymotrypsin-type serine protease. (**B**) The chymotrypsin-type serine protease fold in S3. The putative catalytic triad of CSPro is displayed in stick and colored by elements. The structures in (**B**) and (**C**) are colored in a rainbow scheme with the N terminus in blue. (**C**) Phylogenetic analysis of the CSPro domain. The best-fit model was Q.pfam + I + R5. The support node annotations are the same as those in Fig. 4. The complete tree with all branches is shown in . (**D**) Phylogenetic analysis of the SF2H domain of FaVvV1. The model of substitution was Q.pfam + F + R7. The putative cellular homologs of the SF2H domain and ATP-dependent helicase HrpB were defined as the outgroup. The support node annotations are the same as those in Fig. 4. The complete tree with all branches is shown in Fig. S9. (**E**) Genome diagram of a potyvirus sweet potato mild mottle virus (SPMMV), FaVvV1, and AfVlV1. Dashed lines indicate the homologous domains.

Other vivivirus-encoded proteins, except VP1 and VP2, have not been associated with any known functions, probably due to limited sequence data. For example, when VP3 of FaVvV1 was subjected to a BLASTP search against the NCBI non-redundant database, only one hit was found: a hypothetical protein (32.63% identity and 74% coverage, accession number BED98313.1) of AfVvV1. Here, leveraging diverse sequence data from assembled viral segments, we found that VP3 is present in all assembled viviviruses and vipoviruses but absent in pucciniviruses (Fig. 2; Table S4 and S5). Alignment of VP3 showed conserved motifs distantly related to the nuclear inclusion protein a (NIa) of potyvirids in *Pisuviricota*. Based on the alignment, the 3D structure of VP3 was confidently predicted using AlphaFold2, comprising four structural domains. The first domain (35–295 aa) lacks predicted functional annotation (Fig. 5A). The second (300–510 aa) and third (525–715 aa) domains display structural similarity to a chymotrypsin-type protease with two β-barrels (Fig. 5A). A putative catalytic triad consisting of His^357^, Asp^400^, and Ser^475^ was predicted to be situated between the β-barrel of the second domain, which was then designated as chymotrypsin-type serine protease (CSPro) (Fig. 5B). Although the third domain (CSPro-like) lacks the catalytic triad characteristic of CSPro (Fig. 5B; Fig. S8A), the residues Arg, Asp/Glu, and Asp, which are involved in the formation of putative ionic or hydrogen bonds that may contribute to the stability of the β-barrel, remain conserved (Fig. S8B). These analyses imply that the CSPro-like domain may evolve from duplication of the CSPro in vivivirids, which has also been suggested during the evolution of chymotrypsin (45). Phylogenetic analysis of the CSPro domain indicated that vivivirids, along with the unclassified AfVlV1, clustered with the NIa of potyvirids (Fig. 5C). The fourth structural domain (720–855 aa) of VP3, comprising a six-helix bundle, resembles the RNase III-like core (PF14622) found in the fungus *Mucor lusitanicus* R3B2 (46) (Fig. S8C and D). A similar RNase III domain was also identified in all pucciniviruses (Fig. S8C). Interestingly, another class of RNase III—the mycoviral RNase III domain (PF20614), whose function in mycoviruses remains unknown—was discovered in segment S6 of viviviruses within the papain-like cysteine protease (PCPro) clade and in segment S5 of AfVlV1 (Fig. S3; Fig. S8C and D).

VP5 of FaVvV1 contains an SF2H domain, which is conserved in all vivivirids. Phylogenetic analysis of the viral SF2H domain revealed that vivivirids clustering with jiviviruses were closely related to unclassified mycoviruses, including AfVlV1 (Fig. 5D). Notably, AfVlV1 encodes both SF2H and CSPro domains in a single ORF in its third segment similar to Erysiphe necator-associated poty-like virus 2 and 3 (Fig. 5C and D). These arrangements resemble the CI-NIa domain order of potyvirids, implying a transitional state between potyvirids and segmented vivivirids (Fig. 5E). However, tobamo-like viruses and jiviviruses contain the SF2H domain but lack the CSPro domain, implying that the SF2H domain was obtained through a different evolutionary trajectory or that the CSPro domain was lost in these lineages. Taken together, the results suggest that VP3 and VP5 share common ancestors with their counterparts of potyvirids in *Pisuviricota*, shedding light on the evolution of potyviruses.

### Complex evolution of PCPro domain-containing proteins in viviviruses

**Fig 6:**
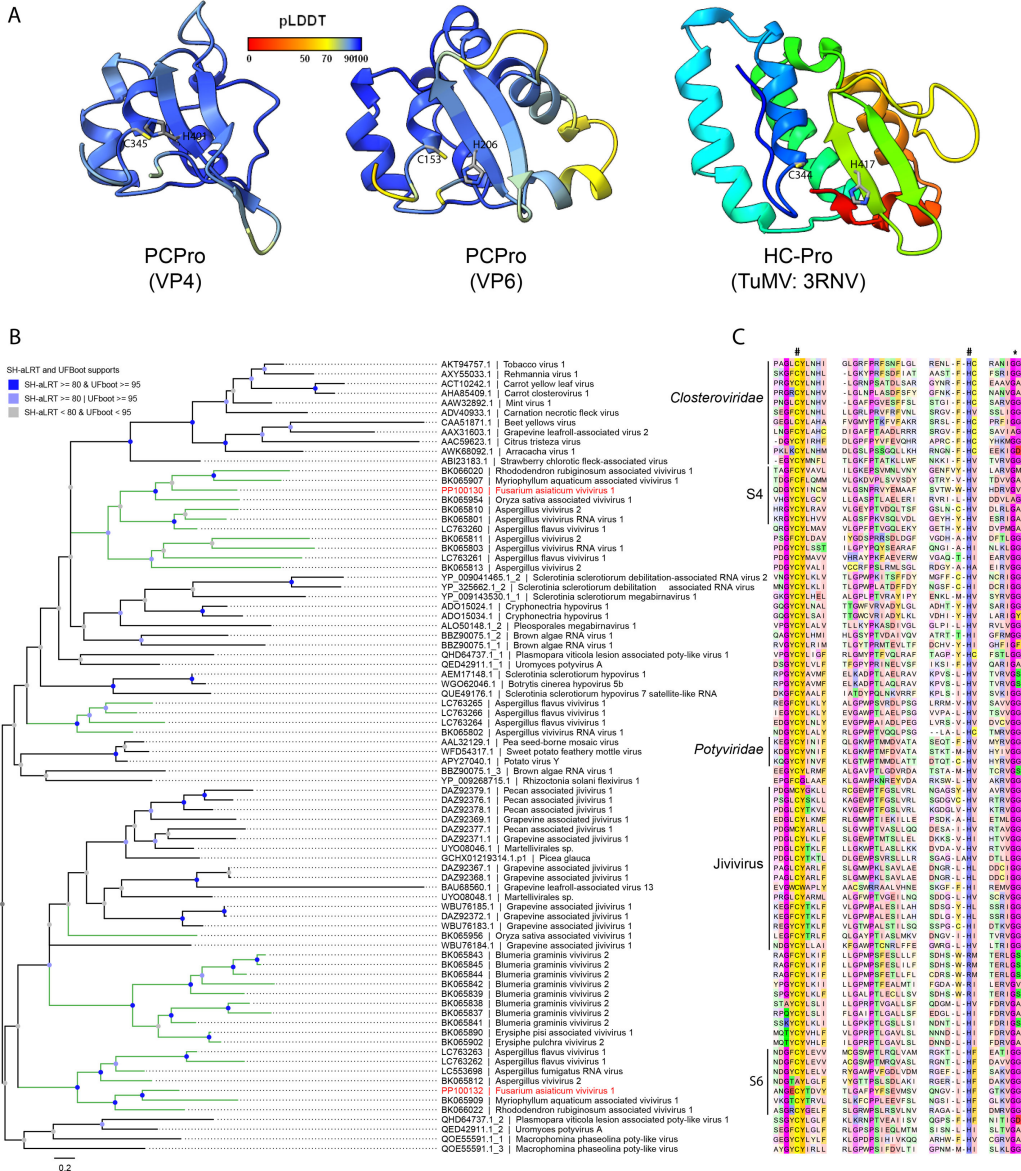
Expansion of papain-like cysteine protease domains in viviviruses. (**A**) The 3D structure of the PCPro domain of VP4 and VP6 as predicted using AlphaFold2. The structure of PCPro in VP4 and VP6 is colored according to the pLDDT value. The putative catalytic triads of PCPro are displayed in stick and colored by elements. The structure of HC-Pro of turnip mosaic virus is colored in a rainbow scheme, with the N terminus in blue. PCPro, papain-like cysteine protease. HC-Pro, helper-component protease. (**B**) Phylogenetic analysis of the PCPro domain. The best-fit model was WAG + F + R4. The annotations are the same as those in Fig. 4. (**C**) Alignment of the PCPro domain. The catalytic dyad and cleavage sites are marked using a number sign (#) and asterisk (*) at the top, respectively. The full alignment is shown in Fig. S8.

The papain-like cysteine protease (PCPro), a subclass of cysteine peptidases similar to papain, is widely found in some clades of +ssRNA viruses infecting animals and plants, such as *Togaviridae* and *Potyviridae*, but is only pervasive in mycoviruses in *Hypoviridae* (47, 48). Here, we discovered two segments of viviviruses, previously predicted to encode hypothetical proteins, that encode PCPro domains. The VP4 and VP6 of FaVvV1 shared 28.87% identity (79% query coverage) with a hypothetical protein (BED98315.1) of AfVvV1 and 28% identity (32% query coverage) with a hypothetical protein (BCH36639.1) of AfRV1, respectively. However, alignment with VP4 and VP6 homologs showed conserved motifs (GXCY…H/R…[VILM]G|G) similar to the PCPro domain of hypovirids and potyvirids (Fig. S11). To further confirm that VP4 and VP6 indeed encoded a PCPro domain, we predicted the 3D structures of the PCPro domain using AlphaFold2. Sequence alignments and 3D structures of PCPro reveal a conserved catalytic dyad (Cys and His/Arg) located at the α-helix and β-sheet, respectively, with a cleavage site ([VILM]G|G) adjacent to the catalytic site (Fig. 6A; Fig. S11 ). Compared to the 3D structure of the helper component-protease (HC-Pro) of potyvirids, the two PCPro domains of FaVvV1, particularly the PCPro in VP4, displayed a shorter and more compact structure (Fig. 6A; Fig. S11). VP4 and VP6 are specific to viviviruses in the “PCPro” subclade and have not been found in other viviviruses or vipoviruses (Fig. 2 and 4). Moreover, some viviviruses within the “PCPro” subclade, such as AfVvV1, AfRV1, Aspergillus vivivirus 2, and Oryza sativa-associated vivivirus 1, have additional segments encoding the PCPro protein (noted as PCPro1 and 2 in Fig. 2B; Fig. S11). However, PCPro domain-containing proteins are rarely found in other viviviruses outside this subclade (6/16) and are entirely absent in vipoviruses (0/8) (Fig. 2B). Surprisingly, Blumeria graminis vivivirus 2 (BgVvV2, Table S4) contains 15 genome segments with nine encoding the PCPro domain-containing protein—three times more than most vipoviruses in terms of genome segment number. All PCPro domains of BgVvV2 clustered together, implying that they probably evolved from gene duplication (Fig. 2 and 6B). Similar duplications occurred in viviviruses, including AfVvV1, AfRV1, and Aspergillus vivivirus 2. Our analysis also indicated that some recently discovered segments of jiviviruses actually encode a PCPro protein, likely evolved through duplication events (Fig. 6B and C) (49). Due to poor support for most deep nodes in the PCPro tree, the relationship between the S4-/S6-encoded PCPro proteins and their counterparts in closterovirids and potyvirids remains unresolved (Fig. 6B). Taken together, our analysis suggests that gene gain/loss and gene duplication within specific clades contribute to the evolution of PCPro proteins in viviviruses.

### FaVvV1 is potentially related to hypovirulence in *F. asiaticum*

**Fig 7:**
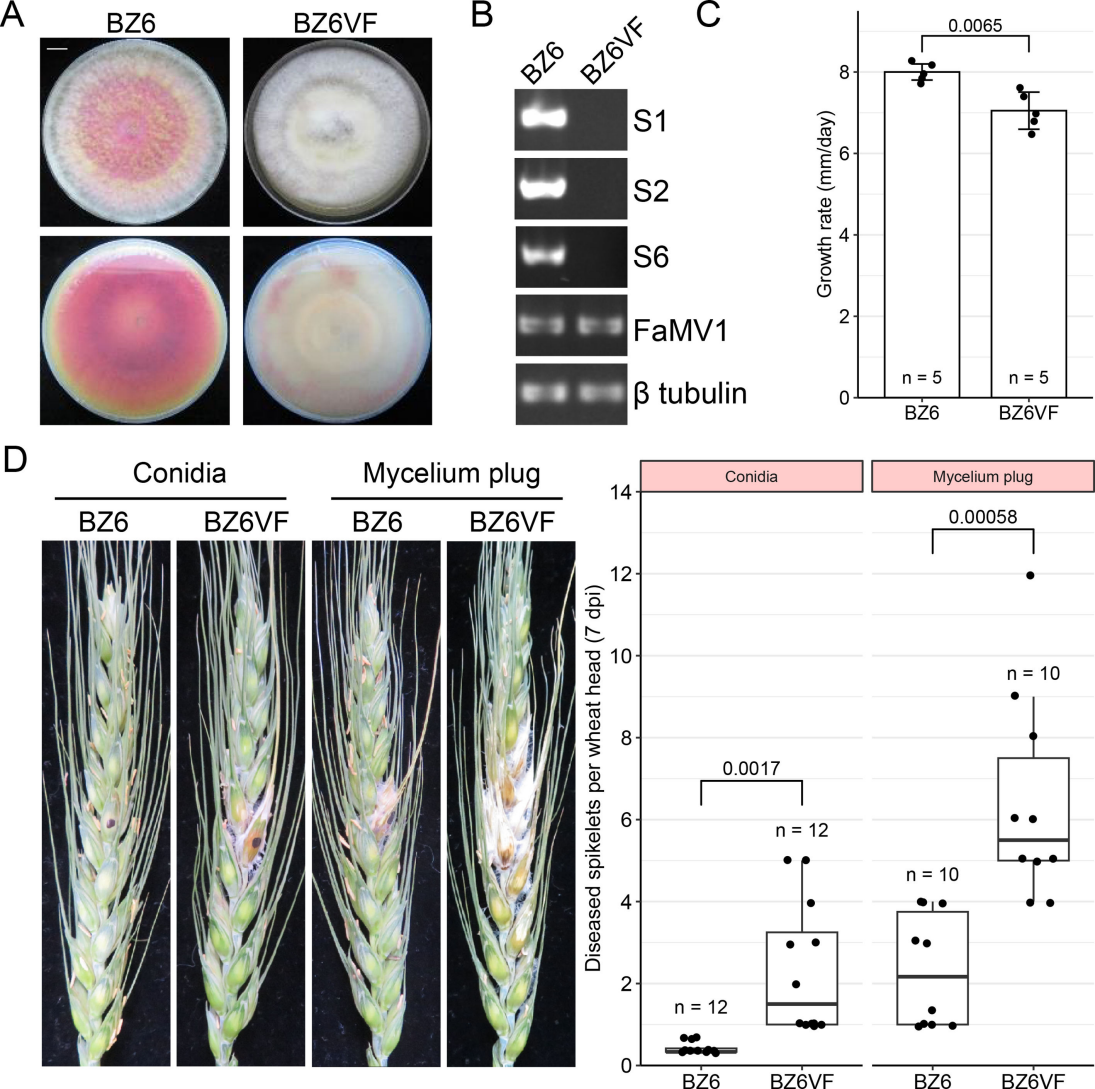
Effects of FaVvV1 on *F. asiaticum*. (**A**) Colony morphology (front and rear of plates) of FaVvV1-related strains. Scale bar, 1 cm. (**B**) RT-PCR detection of FaVvV1 and FaMV1. BZ6, a wild-type strain co-infected with both viruses; BZ6VF, a derived strain of BZ6 with FaVvV1 eliminated. NTC, no template control. (**C**) Growth rates of strains BZ6 and BZ6VF on PDA. Bars represent mean ± SD, and the *P*-value was calculated using an unpaired two-tailed *t*-test. (**D**) Wheat heads of Zhengmai 9023 inoculated with conidia and mycelium plugs of BZ6 and BZ6VF. The inoculated spikelet was marked with black dots. Scale bar, 1 cm. The boxplot displays individual data points (dots), the median (middle line), the 25th and 75th percentiles (box), and the lower and upper whiskers, which extend from the 25th and 75th percentiles to the smallest and largest value within 1.5 × interquartile range (IQR). *P*-value was calculated using unpaired two-tailed *t*-test.

To define the effects of viviviruses on their hosts, we explored the relationship between FaVvV1 and phenotypic changes in *F. asiaticum*. Virome analysis and RT-PCR indicated that strain BZ6 was co-infected with two mycoviruses, FaVvV1 and Fusarium asiaticum mitovirus 1 (FaMV1) (Fig. 7A and B). To assess the biological effects of FaVvV1, we eliminated FaVvV1 from the BZ6 strain via single-spore isolation. The results showed that FaVvV1 could be vertically transmitted via conidia with approximately 75% efficiency (18/24) but was eliminated in all ascospore progenies (0/21) (Fig. S12). Furthermore, both S9 and S10 exhibited instability, with detection rates of 50% (12/24) for S9 and 58.3% (14/24) for S10, which were lower than the 75% (18/24) detection rate observed for FaVvV1 (Fig. S12B and C). However, FaMV1 was not eliminated by either sexual or asexual reproduction (Fig. S12). A representative FaVvV1-free strain (BZ6VF) was selected for subsequent characterization. Compared to the BZ6 strain, the BZ6VF strain showed a decrease in growth rate *in vitro* but enhanced virulence in wheat heads (Fig. 7B through E). Collectively, FaVvV1 could potentially be related to hypovirulent phenotypes in *F. asiaticum*.

## DISCUSSION

FaVvV1 is a 10-segmented +ssRNA virus with rigid rod-shaped virions. Together with the recently discovered AfVvV1 (24), FaVvV1, and other assembled vivivirids in this study indicate that vivivirids exhibit notable genomic diversity, ranging from 5-segmented RNA viruses, like the vipoviruses, to 15-segmented BgVvV2. Genome content and phylogenetic analysis suggest the splitting of vivivirids into two clades: vivivirus and vipovirus. All vivivirids share common genomic features, including the Mtr1-RdRP domain arrangement in S1, the Mtr2-SF1H domain arrangement in S2, the CSPro domain in S3, the SF2H domain in S5, and the capsid encoded by S8. Moreover, acquisition and expansion of PCPro domain-containing segments are prominent in the “PCPro” subclade of viviviruses. These unique features seem to be characteristics of “Viviviridae.” Finally, we provided evidence that vivivirids evolved through multiple genome segmentations and gene/domain duplications and revealed a possible evolutionary relationship between potyvirids and martellivirals.

Although metaviromics has revolutionized virus discovery, determining the complete genome of novel segmented viruses remains challenging. However, by exploiting the conserved and highly similar terminal sequences of segmented viruses, new sequencing techniques and analytical tools have been developed and utilized to deal with this difficult task (24, 50). In this study, by discovering additional genomic segments of viviviruses, we revealed common genome features of vivivirids. However, unique/orphan segments, such as S7, S9, and S10 of FaVvV1, are not detected in other viviviruses. Oddly, unique/orphan segments were scarce in other viviviruses assembled from SRA data. This could be due to overlooked unknown contigs in virus mining or because S7 and S9 (S10) were recently obtained or specifically retained in FaVvV1.

Another concern is determining the actual hosts of the viviviruses assembled from SRA data, which are derived from plant samples colonized by commensal microorganisms, especially phytopathogenic or endophytic fungi. Therefore, these identified vivivirids may infect either plants or phytopathogens, consistent with the previously described viviviruses from *Plasmopara viticola* lesions (14). However, the discovery of AfVvV1 (8, 15, 24) and FaVvV1 implies that fungi are undoubtedly (one of) the hosts of viviviruses. Moreover, some SRA data derived from samples solely containing fungi, such as Synchytrium endobioticum vivivirus 1 (SRR8074753), Cordyceps militaris vivivirus 1 (SRR8640098), Melampsora × columbiana vivivirus 1 (SRR10229823), and Melampsora allii-populina vipovirus 1 and 2 (SRR4063167), further support the viewpoint of fungi as the hosts of vivivirids. Similarly, the host range of “puccinivirus” might also include fungi, as exemplified by Melampsora allii-populina puccinivirus 1, which was assembled from SRA data of an axenic fungal culture. Considering that vivivirids are abundant in plant samples colonized with fungi, and that most relatives of vivivirids are plant or plant-associated viruses, whether plants can serve as hosts remains to be explored. Research on Aspergillus lentulus jivivirus 1 suggests that fungi are also potential hosts for jiviviruses (44). These results unveil the hidden diversity of multiple-segmented +ssRNA viruses in fungi.

Our study provides the first characterization of vivivirus virions. Although previous studies attempted to purify AfVvV1 virions, mixed viral infections and uncertainty in capsid identification led to the characterization of AfVvV1 particles as spherical (15). Here, we observed rod-shaped virions in strain BZ6 co-infected with FaVvV1 and the capsidless mitovirus FaMV1 and identified that the capsid is encoded by the S8 segment of FaVvV1. The CP of FaVvV1 exhibits structural similarities but forms a different cluster compared to CPs of members from the families *Potyviridae* and *Closteroviridae*. This observation appears contradictory to the rod-shaped virion of FaVvV1, considering that the CPs of rod-shaped viruses (*Virgaviridae*) and flexuous filamentous viruses (*Potyviridae* and *Closteroviridae*) are phylogenetically distinct and belong to different clades (51). One possible explanation for our observation is that differences in the β-hairpin and α6 helix may influence the architecture of the virion and the contacts between CPs (52). Because the flexibility of the virion depends on the interaction of CP molecules, it cannot be excluded that a CP that formerly formed a flexuous virion may evolve to build a rod-shaped virion (53). On the other hand, relatively shorter genome segments of viviviruses (<4 kb) could tolerate the lower flexibility of the virion compared to potyvirids and closterovirids. Given the similarities between vivivirid replication proteins (VP1 and VP2) and those of virgavirids, as well as VP8 to potyvirids, and considering that all known segmented viruses in *Virgaviridae* and *Potyviridae* are multipartite (54), we hypothesize that vivivirids may also adopt a multipartite genome organization. Further experiments—such as high-resolution cryo-electron microscopy analysis of FaVvV1 virions or more refined virus particle purification—are required to test this hypothesis.

Our study suggests that a portion of the genome segments of vivivirids evolved via genome segmentation, a process similarly documented in Jingmen tick virus (55). In detail, S1 and S2 evolved from the splitting of a progenitor virus of martelliviral with a conserved Mtr-SF1H-RdRP gene module, and later, a novel Mtr domain (Mtr1) was acquired via HGT or duplication to achieve the Mtr1-RdRP domain arrangement in S1, as was also hinted in previous reports (8, 14). Such evolutionary events may also occur in jiviviruses (43), AfVlV1 (24), and pucciniviruses. Furthermore, the similarity of S3 and S5 in vivivirids to CI and NIa in potyvirids and the retention of SF2H and CSPro domains in a single segment of AfVlV1 provide insights into genome recombination or genome segmentation events in virus evolution. In one evolutionary trajectory, the S3 and S5 segments of vivivirids may have evolved into SF2H-CSPro, as seen in AfVlV1, serving as an intermediate state leading to potyvirids. Alternatively, in another trajectory, these two segments of vivivirids may have arisen through genome segmentation of an ancestral segment with an SF2H-CSPro domain arrangement. The evolution of jiviviruses may represent a distinct scenario: either (i) the genome segment encoding the CSPro domain was lost (or never acquired) or (ii) the SF2H domain-encoding segment originated from a viral lineage different from potyviruses. This hypothesis is supported by the fact that only the SF2H domain-encoding segment has been identified in jiviviruses so far (49, 50). Genome segmentation in vivivirids may represent an evolutionary compromise between genome size/content and virion flexibility. This scenario parallels the fitness advantage conferred by genome segmentation in foot-and-mouth disease virus, where a bipartite genome organization (rescued through complementation between two defective RNAs) outcompetes monopartite genomes via enhanced virion stability (56). In contrast, two artificially segmented virus variants—a tripartite tomato apex necrosis virus and a four-segmented Rift Valley fever virus—exhibit reduced fitness compared to wild-type viruses (57, 58). Given the large diversity of vivivirids in fungi, their segmented genomes may confer a potential advantage in these hosts.

The evolutionary trajectory of vivivirids presented here indirectly supports the opinion that horizontal virus transfer (HVT) occurring between different hosts plays a central role in RNA virus evolution (59, 60). HVT affords viruses with opportunities for gene exchanges (e.g. recombination, reassortment, and HGT), and once HGT events between viruses are observed, an HVT event probably occurred. Numerous HGT events between viruses have been reported, but an alternative hypothesis is that some “homologous” viral proteins may arise due to convergent evolution (47, 61–65). A relevant example is the evolution of a clade of plant ourmiaviruses, which combined the movement protein and capsid from plant tombusiviruses in *Kitrinoviricota* with the RdRP from fungal botourmiavirus in *Lenarviricota* (61). Although no experimental evidence of HVT being provided, recent discoveries in cross-kingdom transmission of viruses (i.e. HVT) between fungi and plants may consolidate the opinion of HGT events in fungal botourmiavirus and plant tombusivirus (66). For vivivirids, the discovery of tobamo-like viruses and potyviruses in fungi implies the potential for HVT between both martellivirals and potyvirids in both plants and fungi (67, 68), which may be the foundation for the distinctive genomes of vivivirids observed in our study.

Our findings extend the previously known distribution of PCPro domains from *Togaviridae* and *Closteroviridae* to additional lineages within the order *Martellivirales*. The PCPro domains are widespread in viviviruses, especially in the “PCPro” subclade of viviviruses encoding VP4 and VP6, but are absent in vipoviruses, pucciniviruses, as well as AfVlV1 (24), suggesting that different gene gains and losses, and gene duplications are contributing to the diversity of PCPro in segmented fungal +ssRNA viruses. Phylogenetic analysis of the PCPro domain offers only limited support for the hypothesis that vivivirids acquired this domain from potyvirids despite its widespread distribution among potyvirids (69). The PCPro and the RNase III domains may be used to counter the host viral defense by viviviruses, as suggested in previous reports (70–73).

Taken together, our findings reveal the complex diversity of segmented +ssRNA viruses, including vivivirids, pucciniviruses, and previously reported jiviviruses. The vivivirids probably evolved through multiple genome segmentations and gene/domain duplications. The homology of some segments of vivivirids to potyvirids reveals an ancient evolutionary relationship in the evolution of non-segmented and segmented viruses.

## MATERIALS AND METHODS

### Fungal strains and culture conditions

The strains of *Fusarium* spp. were isolated from diseased wheat heads with FHB symptoms in the Sichuan Basin, the middle and lower reaches of the Yangtze River, and the Huang-Huai-Hai region in China. Molecular identification was performed using PCR with the *Fusarium graminearum* species complex (FGSC)-specific primers listed in Table S6 (28), and universal primers ITS1/ITS4 and EF1T/ EF2T were used for *Fusarium* spp. strains outside the FGSC. All *Fusarium* spp. strains were cultured on PDA at 25°C in the dark and stored at 4°C on potato dextrose agar (PDA) slants.

### Vertical transmission of mycovirus in *F. asiaticum*

Strains were cultured in carboxymethyl cellulose medium or mung bean soup medium at 25°C for 5**–**7 days at 180 rpm to produce conidia, and conidial suspensions were obtained by filtering, centrifuging, and resuspending the culture. Single conidium was isolated onto fresh agar medium using a glass capillary needle under a microscope. The germinated conidia were then transferred to PDA plates to obtain single-conidium progenies. Sexual development of *Fusarium* sp. was carried out as previously described (74), and single-ascospore progeny was obtained using the same single-spore isolation method described above. Total RNA of single conidium/ascospore progeny was extracted, and FaVvV1 and FaMV1 were detected by RT-PCR using mycovirus-specific primers (Table S6).

### Phenotypic measurements of *F. asiaticum*

The biological characteristics of the strains in this study, including growth rate, morphology, and virulence, were assessed as previously described (74) with some modifications. Mycelial growth rate was measured as the distance grown on PDA at 25°C from 24 h to 48 h in four orthogonal directions, and the colony morphology was photographed at 7 days after inoculation. For plant infection assays, detached flowering wheat heads of Zhengmai 9023 were inoculated with 10 µL of conidial suspension (2 × 10^5^ /mL) or a mycelium plug (6 mm) and then bagged to maintain high humidity. Disease symptoms were recorded 7 days post-inoculation. Statistical analyses were performed using Student’s *t*-test.

### Total RNA extraction and sequencing

Fungal strains were cultured on PDA plates overlaid with cellophane membranes for 3–5 days. Approximately 1 g of mycelium was collected and ground to a fine powder in liquid nitrogen. Total RNA was extracted using the NI-Sclerotinia sclerotiorum RNA Reagent (NewBioIndustry, Shachuan [Tianjin] Biotechnology Co., Ltd.) according to the manufacturer’s instructions and treated with DNase I. For metatranscriptomics virus discovery, total RNA from different strains was mixed in equal quantities, and rRNA was removed using a Ribo-zero rRNA Removal Kit (Illumina, San Diego, CA, USA). A Hi-Seq LncRNA-Seq library was constructed for sequencing on the Illumina HiSeq X platform.

### Fungal virus confirmation and full-length determination

To verify the presence of viruses in strain BZ6, cDNA was synthesized using Moloney murine leukemia virus transcriptase (M-MLV; Takara Dalian, China) and random primers (hexa-deoxyribonucleotide mixtures; Takara Dalian, China). Terminal sequences of fungal viruses infecting strain BZ6 were obtained using classic rapid amplification of cDNA ends (RACE) (75) and RNA ligase-mediated RACE methods (76). The primers used for fungal virus confirmation and full-length determination are listed in Table S6.

### Assembly and identification of related viviviruses from selected SRA database

To identify SRA data containing vivivirus-related viruses, the RdRP sequences of FaVvV1 were searched against SRA data in Serratus using palmID (BLAST for Serratus) (40, 77). To make the virus segment assignments easier, only SRA data with high vivivirus-like virus abundance (coverage of the palm >5,000 in palmID results) were considered, and SRA runs with multiple similar viviviruses were filtered out based on the RdRP Search in Serratus Explorer (40). The data were fetched and converted using the SRA toolkit, and the quality of fastq files was evaluated using FastQC v0.11.9; Trimmomatic (version 0.36) (78) or cutadapt (version 4.4) (79) was used for adapter trimming and low-quality read filtering. Subsequently, clean reads were assembled using the SPAdes genome assembler in rnaviral mode and/or Trinity v2.7.0 (80, 81). The assembled contigs were translated using TransDecoder.Long in TransDecoder 5.7.0 and searched against the clustered nr database using mmseqs2 (82). The unannotated contigs were further searched against the hhsuite database, uniprot_sprot_vir70_Nov_2021, and pdb70_from_mmcif_2023–06-18 using HHblits (83, 84). Virus contigs were manually selected, and putative vivivirus segments were confirmed by aligning the 100 bp region of the 5′ terminal ends of the positive strand of contigs with ClustalW or MUSCLE in MEGA-X (85). Finally, all putative segments of the putative viviviruses were clustered using the easy-cluster mode of mmseqs2 (82), and each cluster was aligned to build an hmm database for hmmsearch (HMMER 3.3.2; http://hmmer.org/) to identify missed segments. For orphan contigs, high k-mer coverage contigs (based on the SPAdes results) were selected, and their 5′ terminal ends of the positive strand were aligned with those of the corresponding viviviruses to assess conservation. Reads were mapped to candidate viviviruses using Bowtie2 (version 2.5.1) to exclude chimeric contigs (86). SeqKit was used for sequence processing (87).

### Virus genome annotation and phylogenetic analysis

For viral genomes without functional annotation or domains, we first aligned homologous segments of all assembled viviviruses using muscle 5 in super mode (88). The alignments were then used as the input for HHpred to improve sensitivity. For phylogenetic analysis, similar or homologous protein sequences were obtained using BLASTP and hmmsearch (HMMER 3.3.2) against the nr database or clustered nr databases, respectively. Next, manually curated protein domain alignments were used to build hmm files with hmmbuild, the hits of BLASTP or hmmsearch were aligned to the hmm file by hmmalign (HMMER 3.3.2) to obtain the protein domain, and short sequences were removed using esl-alimanip (HMMER 3.3.2). The easy-cluster mode of mmseqs2 was used to remove highly similar sequences (82). The sequences were then aligned by muscle 5 in super mode (88). Alignments were trimmed using ClipKIT in smart-gap mode (89), and the tree was inferred using IQ-TREE 2.2.6 (90). The R package phangorn 2.11.1 was used to perform the midpoint-rooting of the tree (91), and ggtree 3.10.0 was used for tree visualization (92).

### Virion purification and observation

The BZ6 strain was cultured in six 250 mL shake flasks containing 100 mL PDB medium (supplemented with 1% peptone and 0.5% NaCl) at 25°C and 110 rpm for 5 days. Approximately 60 g wet weight of mycelium was harvested using two layers of cheesecloth and homogenized in 200 mL of 0.01 M PBS using a blender. Mycelial lysates were centrifuged at 15,000 **×** *g* (4°C) to remove mycelial debris. The supernatant was then precipitated through a 20% sucrose cushion at 28,000 rpm (SW32 Ti rotor) for 3 h at 4°C. The pellet was resuspended in 0.01 M PBS and clarified at 12,100 rpm (4°C). The supernatant was separated through a sucrose gradient (20%–60%) at 35,000 rpm (SW 41 rotor) for 3 h at 4°C. After centrifugation, each sucrose layer was slowly aspirated with a blunt pipette, and virions were pelleted at 30,000 rpm (SW32 Ti rotor) to remove sucrose. The virion pellet was resuspended for SDS-PAGE and TEM analysis.

### SDS-PAGE and viral structural protein identification by mass spectrometry

Purified virions were heated and denatured in loading buffer, separated by SDS-PAGE, and stained with Coomassie Brilliant Blue. Bands were cut and subjected to in-gel trypsin digestion. Peptides were identified using Q Exact HF-X (Thermo Fisher Scientific). Proteins translated *in vivo* from all assembled contigs were used as the search database in MaxQuant (v2.4.7.0) (93). Predicted proteins of S8 initiated at the second and third start codons (AUG) were also included in the database to determine the actual translation start site.

### 3D structure prediction, similar structure search, and alignment

ColabFold v1.5.5 was used to predict the 3D structure of FaVvV1 proteins (94, 95). To improve the precision of the predicted structure, a customized multiple sequence alignment was provided, which was obtained by aligning sequences assembled from the SRA data and hits from hmmsearch (HMMER 3.3.2) against the nr database or clustered nr database. Only one of the top-ranked structures was relaxed using amber for runtime considerations. Default settings were used for other options. The predicted structure was searched against the available database in 3Di/AA mode using Foldseek online tools (96). The hits with the highest scores were further compared with FaVvV1 proteins using TM-align (version 20170708) in PyMOL (97). Structures were prepared for publication using UCSF ChimeraX (98).

## Data Availability

Nucleotide sequences of Fusarium asiaticum vivivirus 1 and Fusarium asiaticum mitovirus 1 are available under accessions PP100127–PP100136, and OQ597850, respectively. NGS information is available under BioProject PRJNA1062113. Sequence information of assembled viruses from SRA data is available under accessions BK065799–BK066060. The MSA file, tree files, and PDB files in the study are available at https://doi.org/10.6084/m9.figshare.25922182.v3.

## SUPPLEMENTAL MATERIAL

Supplemental figures jvi.00332-25-s0001.pdf Figures S1 to S12.

Supplemental tables jvi.00332-25-s0002.pdf Tables S1 to S6.

ASM does not own the copyrights to Supplemental Material that may be linked to, or accessed through, an article. The authors have granted ASM a non-exclusive, world-wide license to publish the Supplemental Material files. Please contact the corresponding author directly for reuse.
