# Supplementary material for: A group of segmented viruses contains genome segments sharing homology with multiple viral taxa
Source: J Virol. 2025 Jun 4;99(7):e00332-25. doi: 10.1128/jvi.00332-25 (PMC12282112; doi:10.1128/jvi.00332-25)

Fig. S1. (A) All 10 segments of FaVvV1 detected in 30% sucrose gradient by RT-PCR. (B) MS/MS spectrum of identified peptide segment near the N-terminus of S8. The peptide segment is highlighted in red color below the spectrum, and the putative translation initiating methionine is indicated by an arrow. (C) The globular domain with seven α-helices and one β-hairpin in CP of FaVvV1.

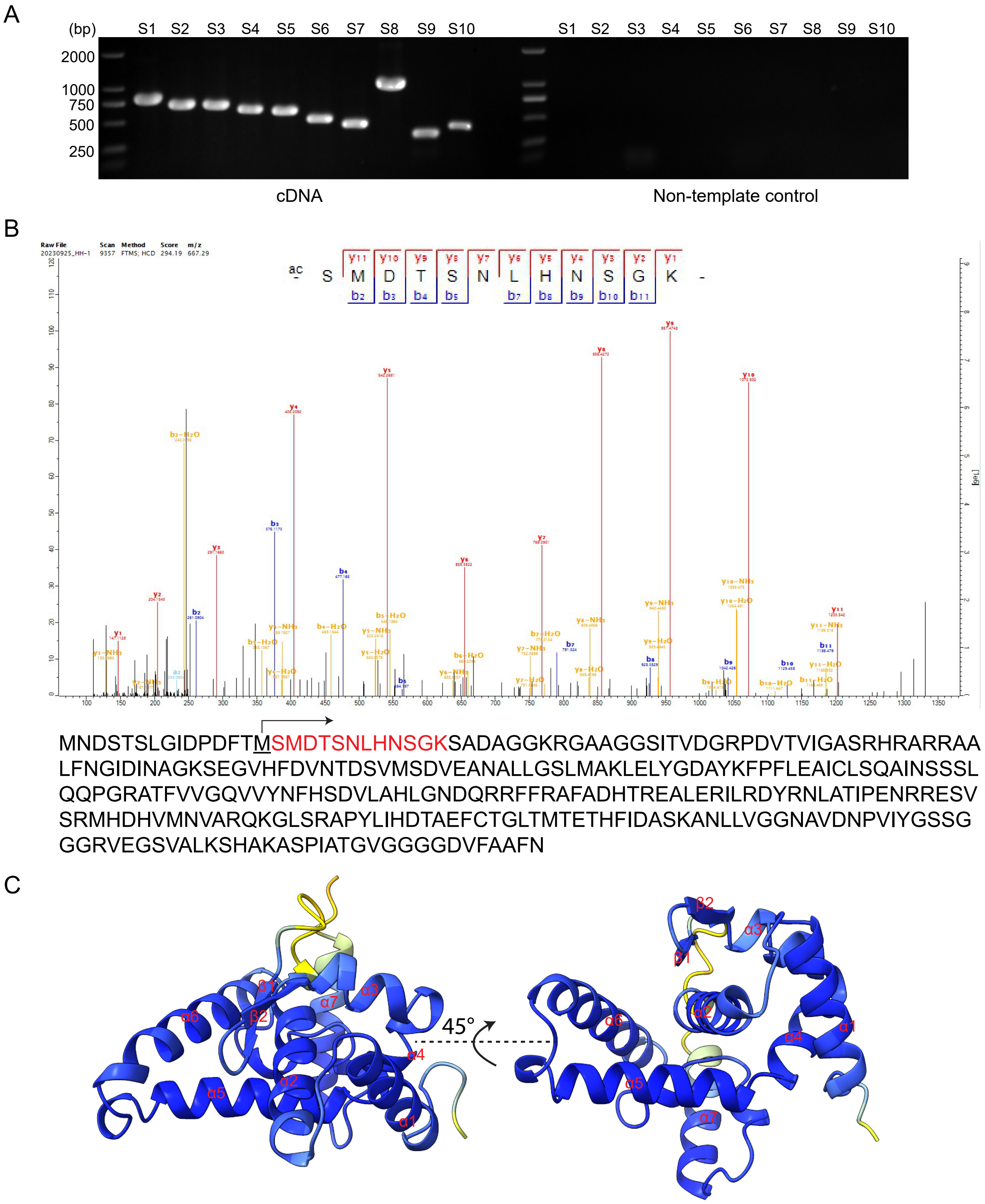

Fig. S2. Pairwise alignment of the sequence (A) and structure (B) of CP by FATCAT2. The representatives of the discovered viruses in this study is highlighted in red.

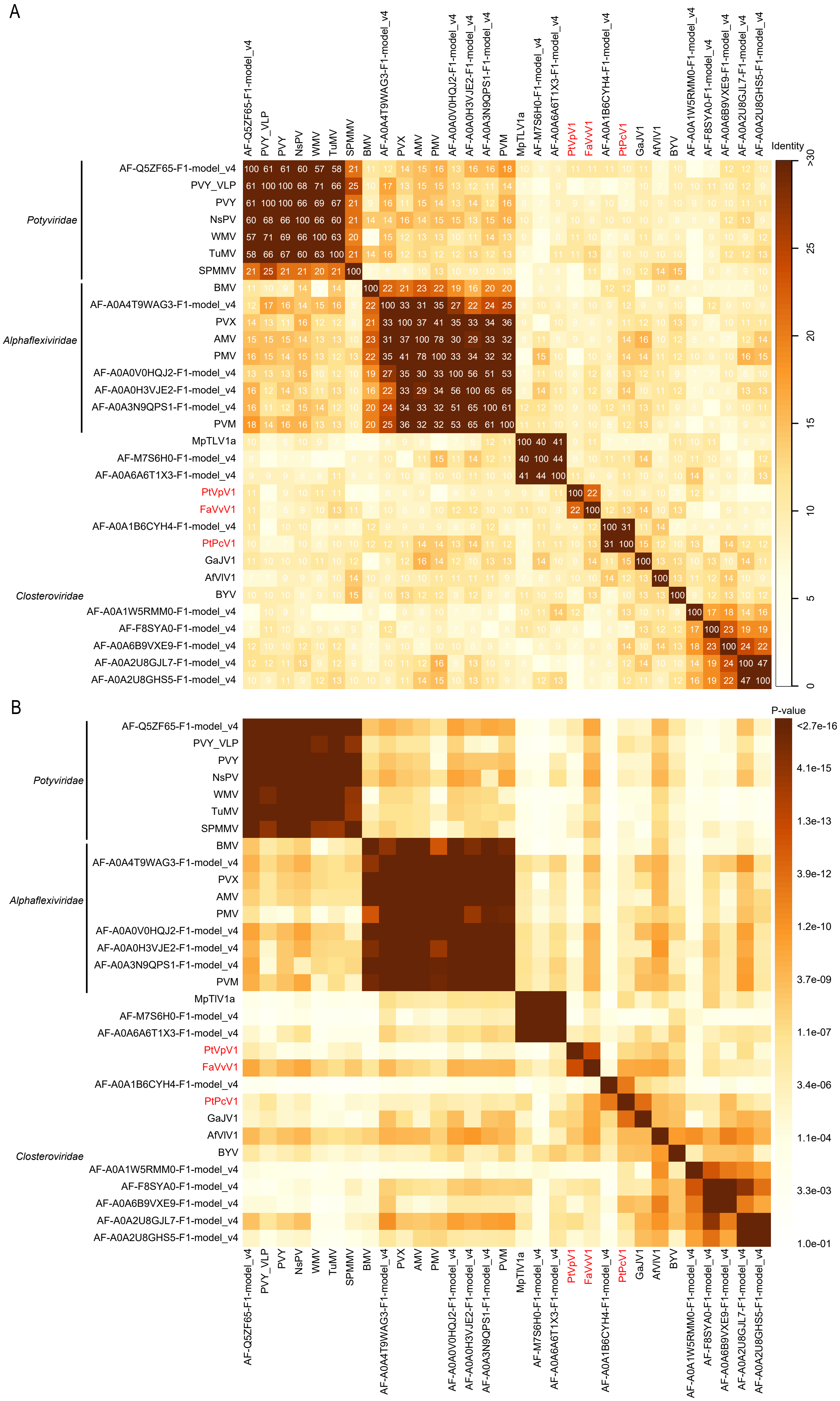

Fig. S3. (A) Genome diagram of vivivirus, vipovirus, AfVlV1, jivivirus and puccinivirus. (B) Best hit of puccinivirus proteins searched by HHpred tool against uniprot_sprot_vir70 and pdb70 databases.

A

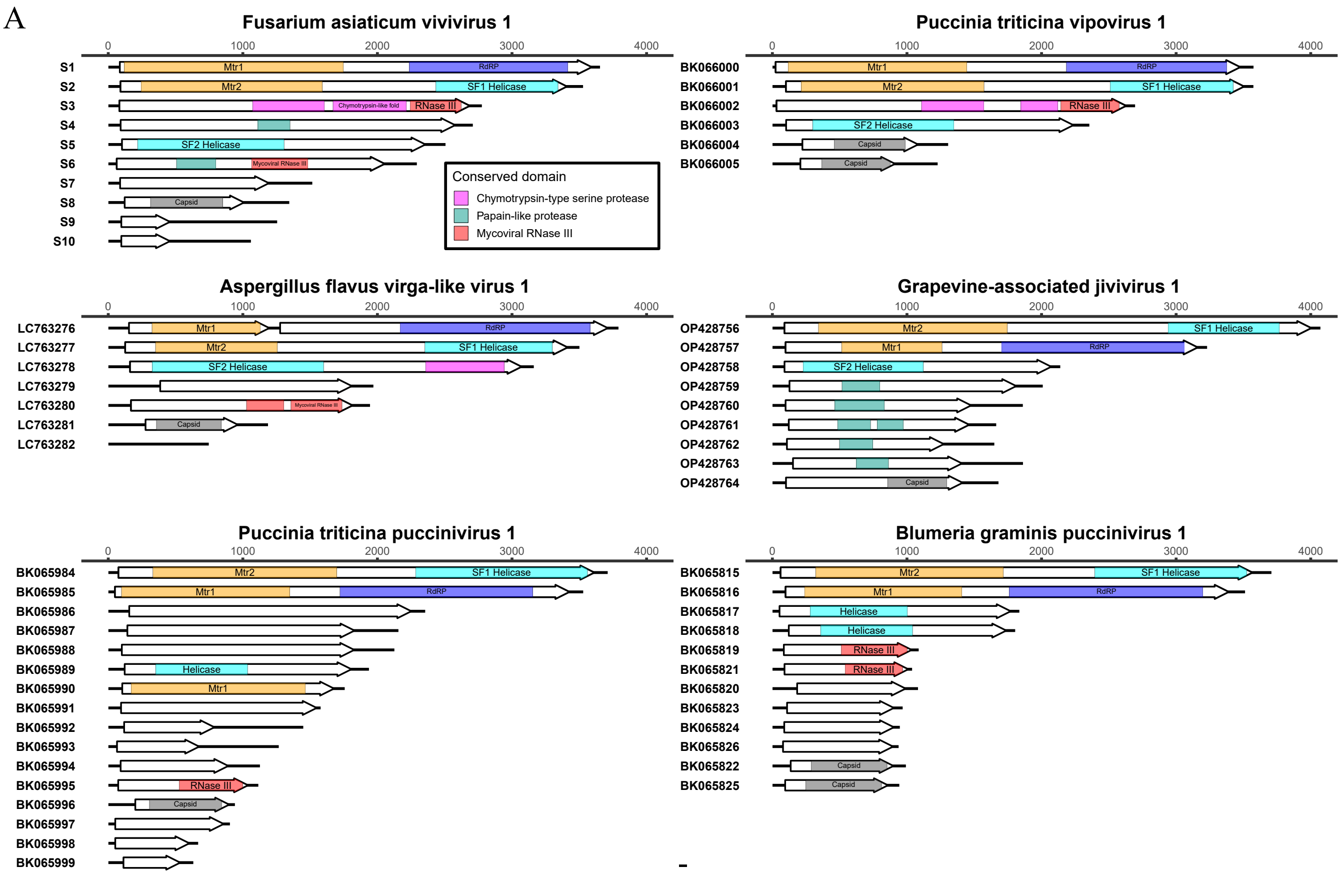

B

Unknown helicase of puccinivirus

No 1

>7OJ4_A NS3 helicase domain; RNA helicase, hydrolase, non-structural protein, VIRAL PROTEIN; 1.828A {Tick-borne encephalitis virus}

Probab=98.95 E-value=2.7e-07 Score=94.97 Aligned_cols=223 Identities=13%

Similarity=0.049 Sum_probs=146.2 Template_Neff=10.800

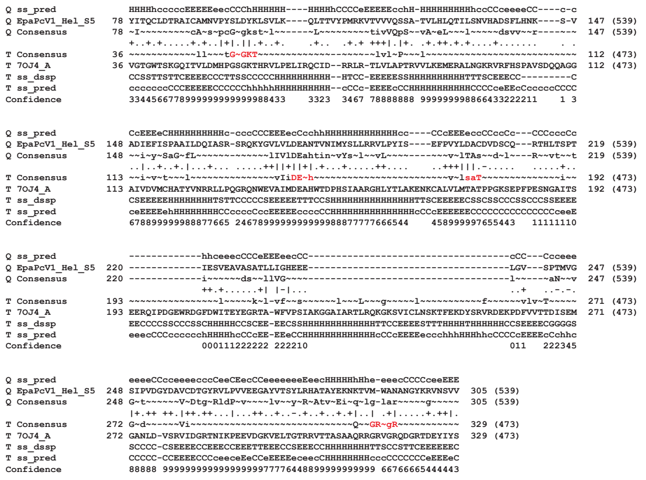

RNase III of puccinivirus

No 1

>7R97_B Ribonuclease 3; protein-RNA complex, HYDROLASE-RNA complex; HET: EDO, TRS; 1.804A {Escherichia coli (strain K12)}

Probab=98.74 E-value=6.4e-07 Score=74.88 Aligned_cols=126 Identities=16%

Similarity=0.164 Sum_probs=101.7 Template_Neff=11.700

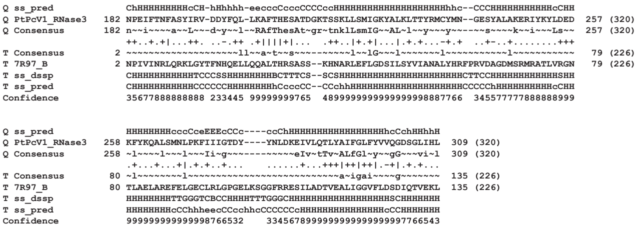

Capsid of puccinivirus

No 1

>P07699 CAPSD_PVX Coat protein OS=Potato virus X OX=12183 PE=3 SV=2

Probab=96.28 E-value=0.071 Score=50.99 Aligned_cols=141 Identities=13%

Similarity=0.161 Sum_probs=0.0 Template_Neff=5.600

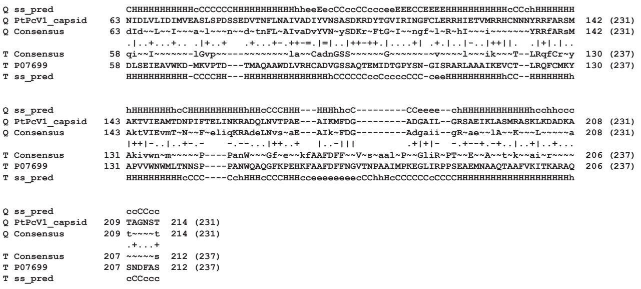

Fig. S4. HMM-HMM alignment of methytransferase domain of S1 and S2 in viviviruses by HHalign 3.3.0 in HH-suite3. Alignment of Mtr1 was aligned to alignment of Mtr2. (A) Mtr domains of FaVvV1 were selected as representative sequences in query (Q) and template (T) sequences. (B) Consensus sequences of the alignment of Mtr1 and Mtr2 of viviviruses calculated by HHconsensus 3.3.0 were selected as representative sequences in query (Q) and template (T) sequences to determine the similarity of the two alignments.

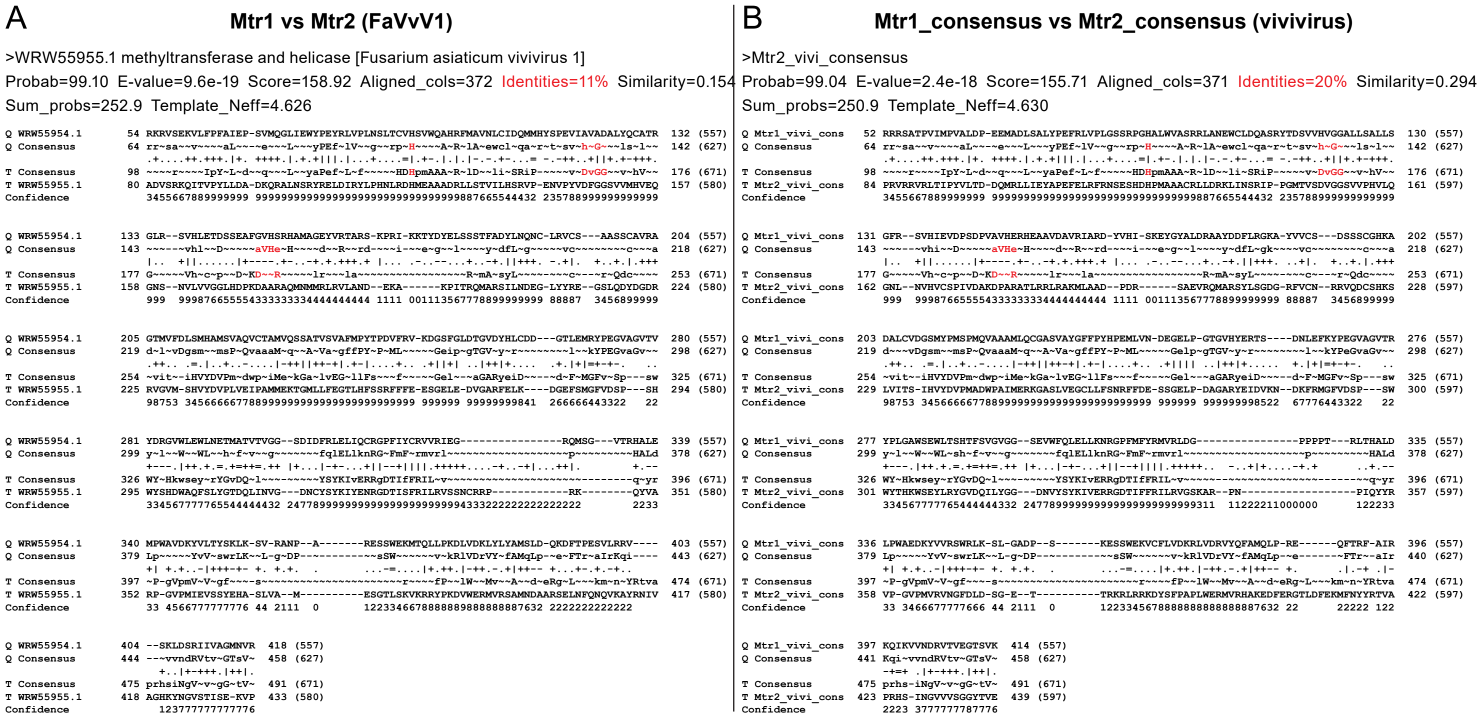

Fig. S5. Alignment of Mtr domain in viviviruses, vipoviruses, jiviviruses, and pucciniviruses. The full name of virus abbrevivation was same in table S1 and table S2. PcV, puccinivirus; Jivi, jivivirus. Vivi, vivivirus. The conserved motif of Mtr domain as shown in Fig. 3A was hilighted in red box.

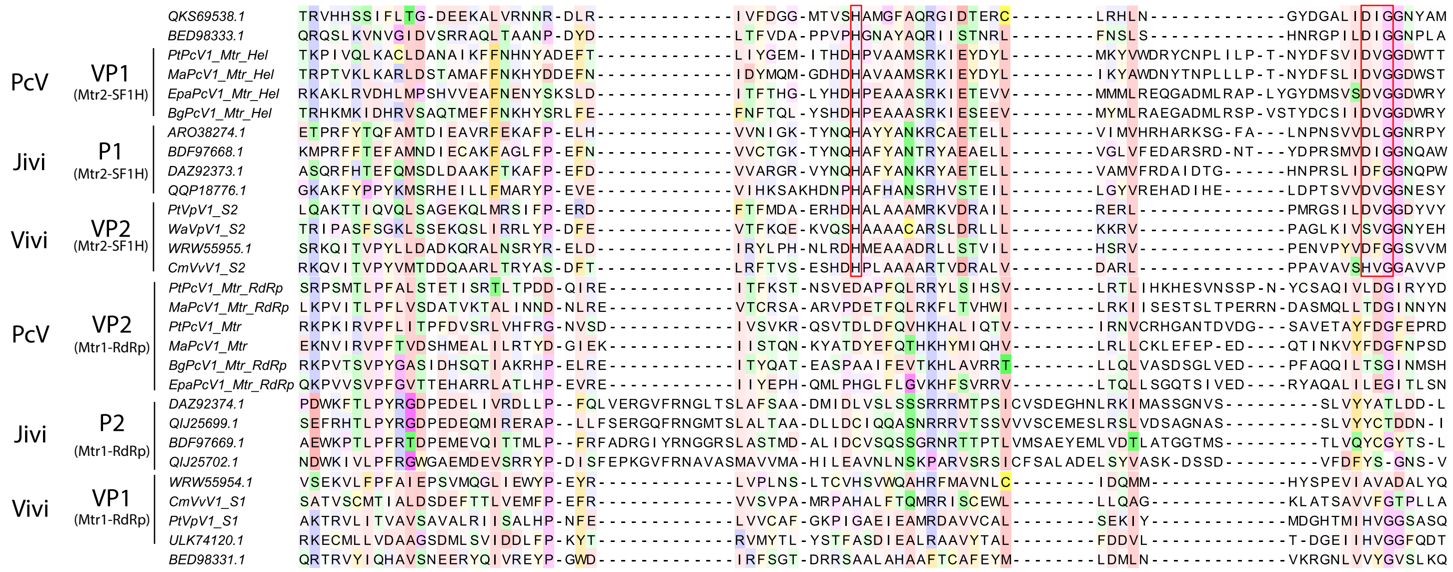

(See next page)

（continue）

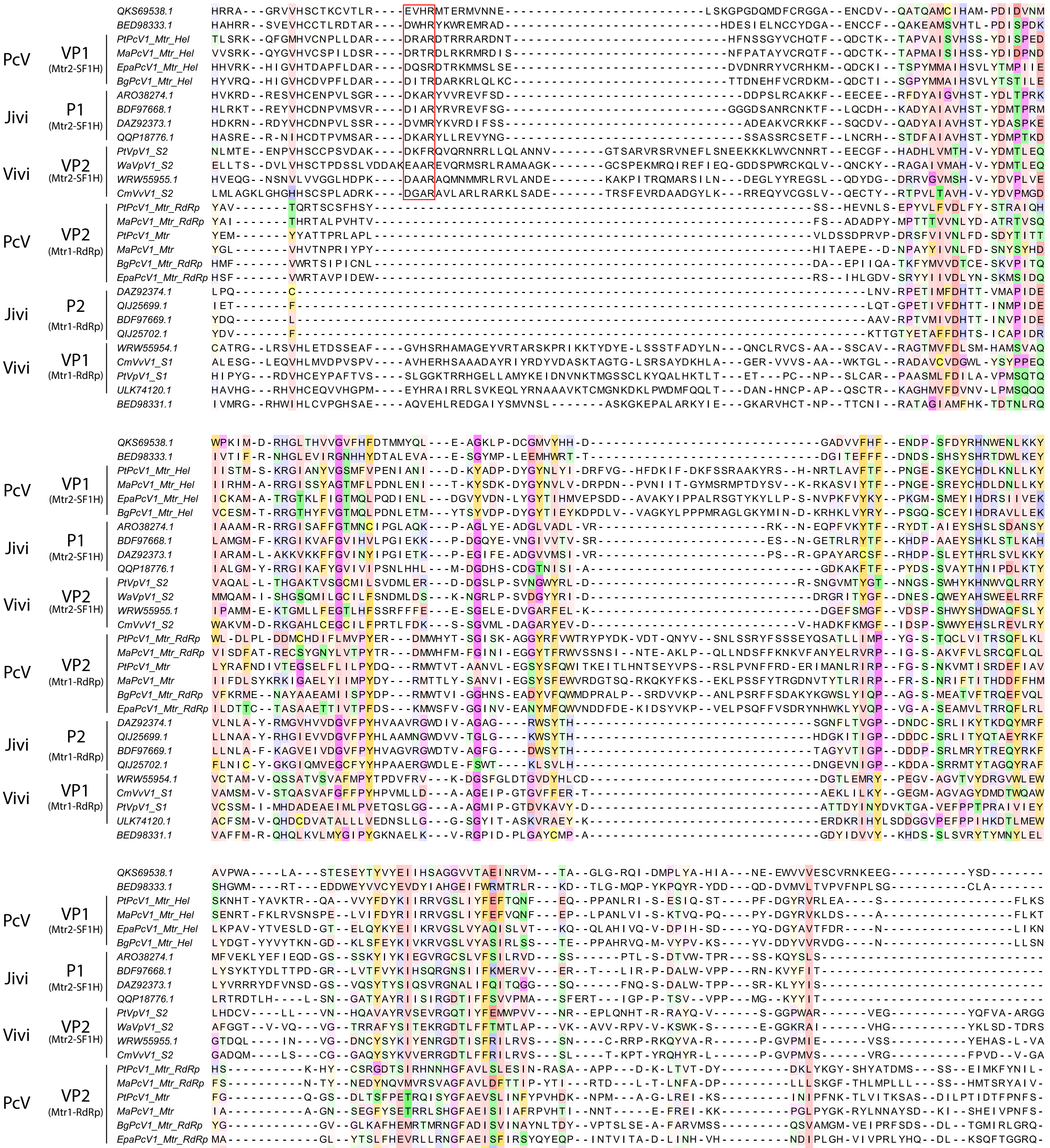

（See next page）

（continue）

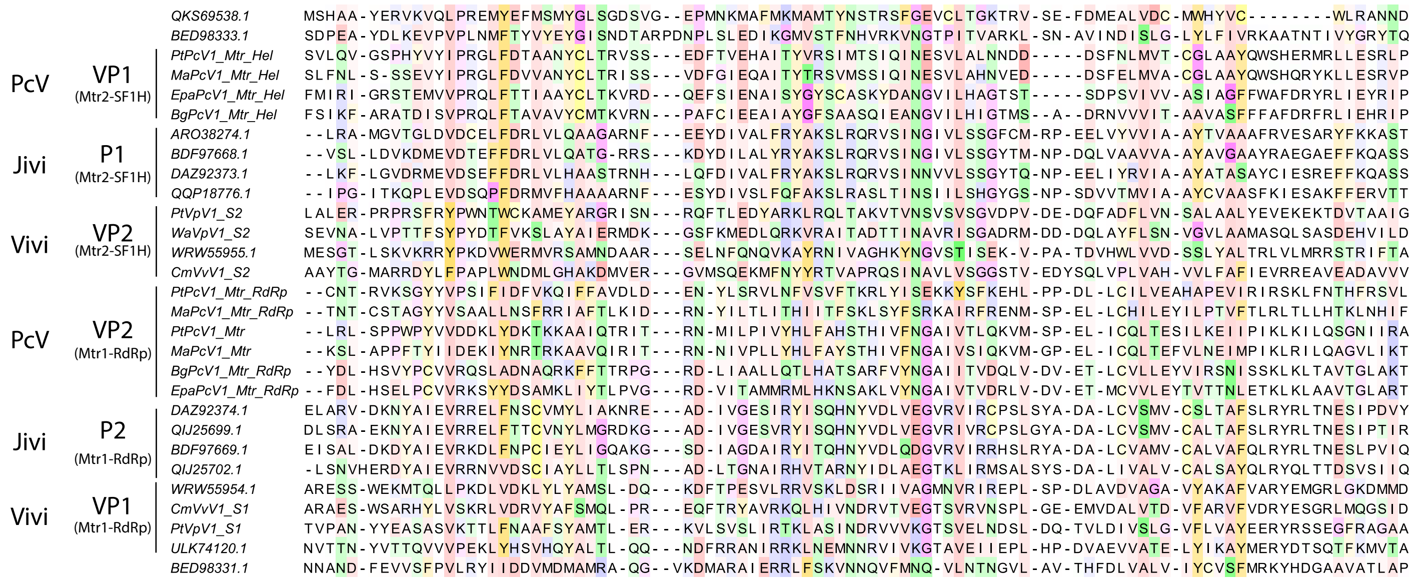

Fig. S6. Homologs detection of N-terminal of S2 (Mtr1-RdRp) in pucciniviruses and jiviviruses by HHpred. The N-terminal of S2 (Mtr1-RdRp) of pucciniviruses and jiviviruses was aligned by Muscle 5, respecitvely, and used as the input, searched against database uniprot_sprot_vir70, NCBI_CD, and pdb70. (A) The first hits of puccinivirus's alignment. PtPcV1 was selected as representative sequences in query (Q) sequences. (B) The fourth hit of jivivirus's alignment was displayed here for the nearly full coverage. Aspergillus lentulus jivivirus 1 was selected as representative sequences in query (Q) sequences. The conserved motif putatively involved in methyltransferase was highlighted in read font.

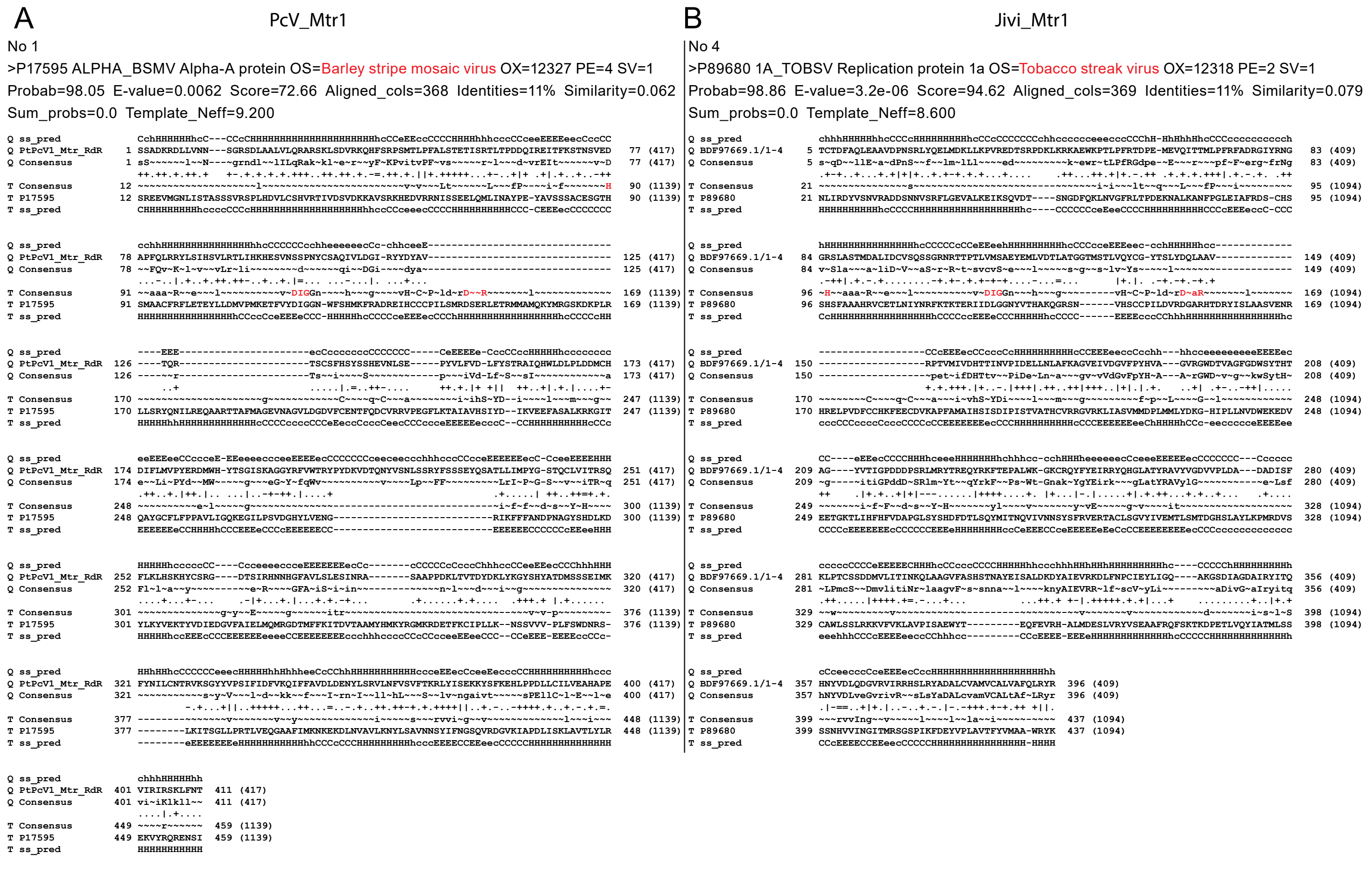

Fig. S7. Phylogenetic tree in Fig. 4 with detailed information. The SH-aLRT / UFBoot support values was labeled at the branch point.

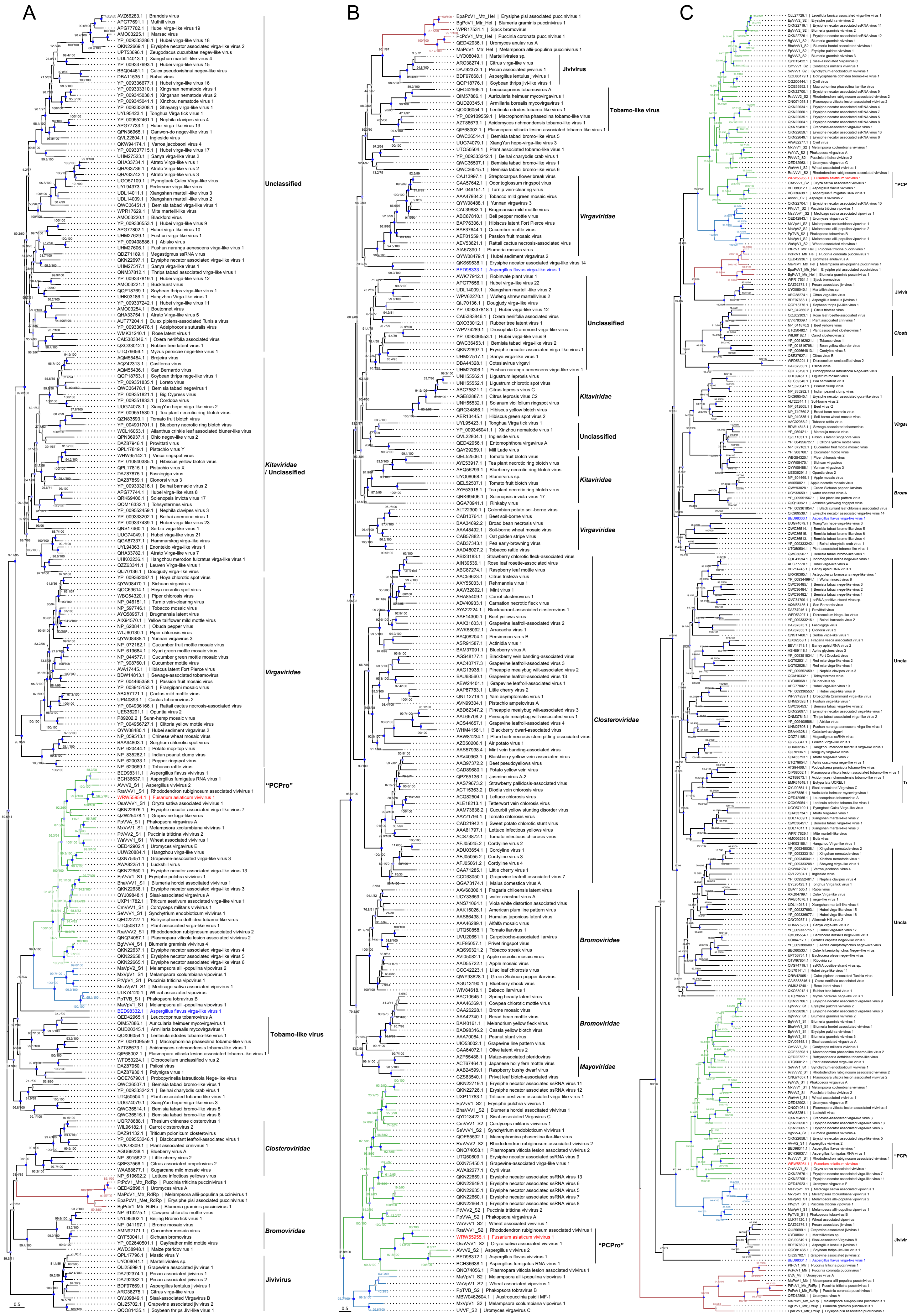

Fig. S8. (A) Alignment of CSPro and CSPro like domain of S3 in vivivirids. The putative catalytic triad His, Asp, and Ser of CSPro are highlighted at the top of the alignment. The conserved residues between CSPro and CSPro like domain involved in hydrogen bond formation were marked with line segments. (B) The chymotrypsin-type serine protease fold in VP3. The conserved residues of CSPro and CSPro like domain were displayed in stick and colored by elements, and the putative hydrogen bond was in dashed line. (C) Alignment of the RNase III domain in VP3 and VP6 of FaVvV1. The putative active sites are highlighted in red box according to structure alignment with R3B2. (D) The structures of RNase III domain in VP3 and VP6 of FaVvV1. The putative active sites are highlighted in stick according to R3B2. All structures in Fig. S6 are colored in rainbow scheme with N terminus in blue.

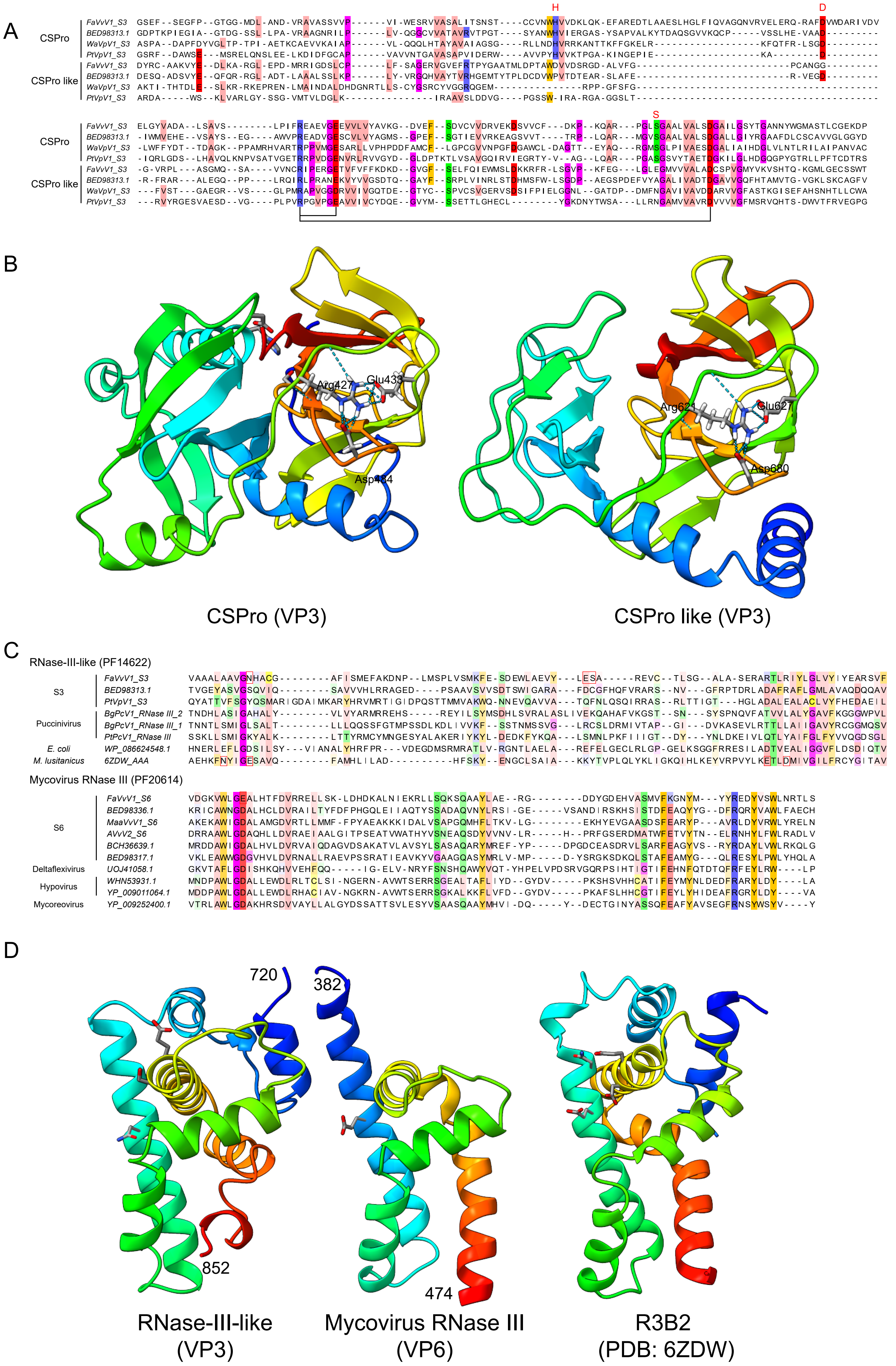

Fig. S9. Phylogenetic tree in Fig. 5C with detailed information. The SH-aLRT / UFBoot support values was labeled at the branch point.

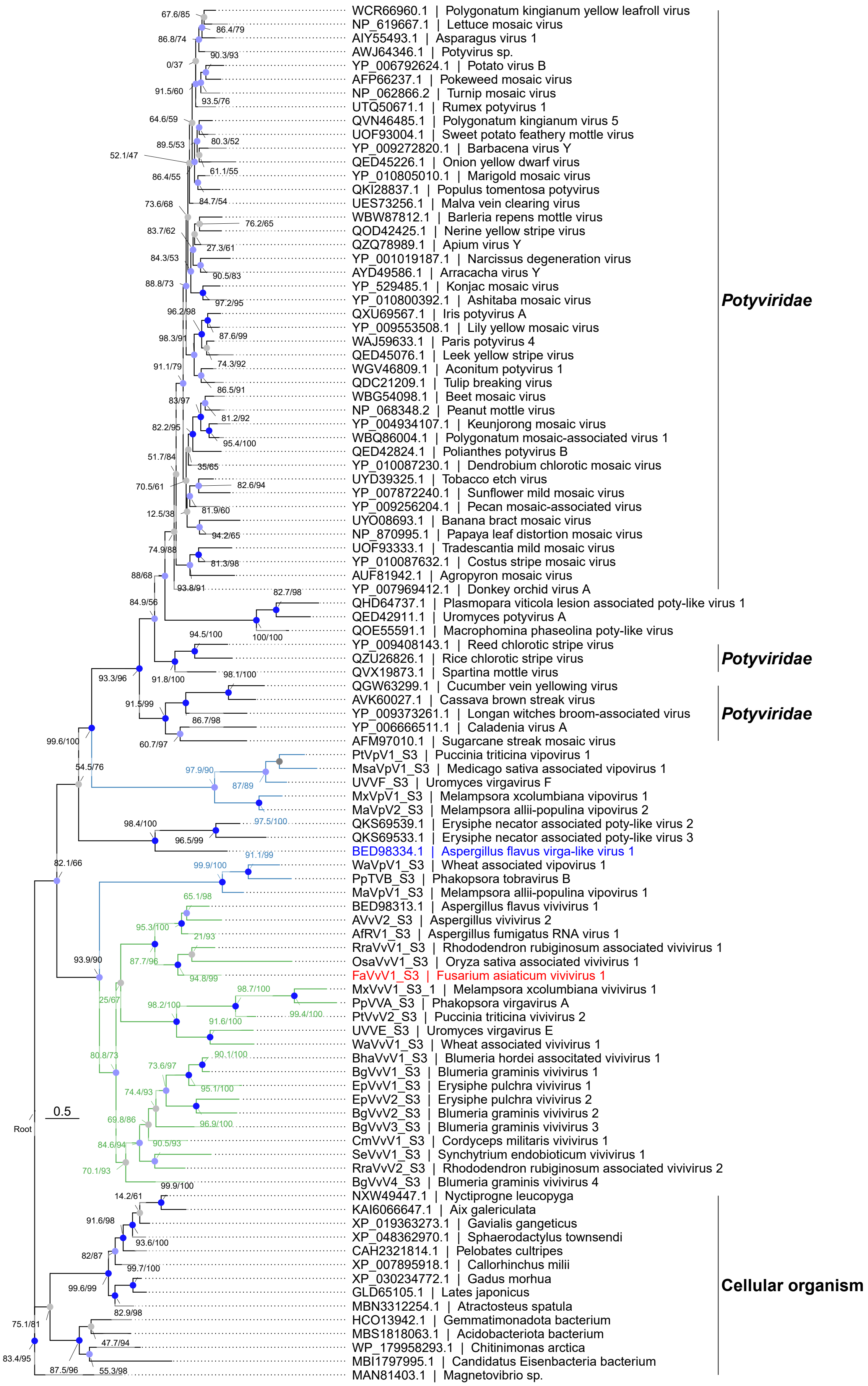

Fig. S10. Phylogenetic tree in Fig. 5D with detailed information. The SH-aLRT / UFBoot support values was labeled at the branch point.

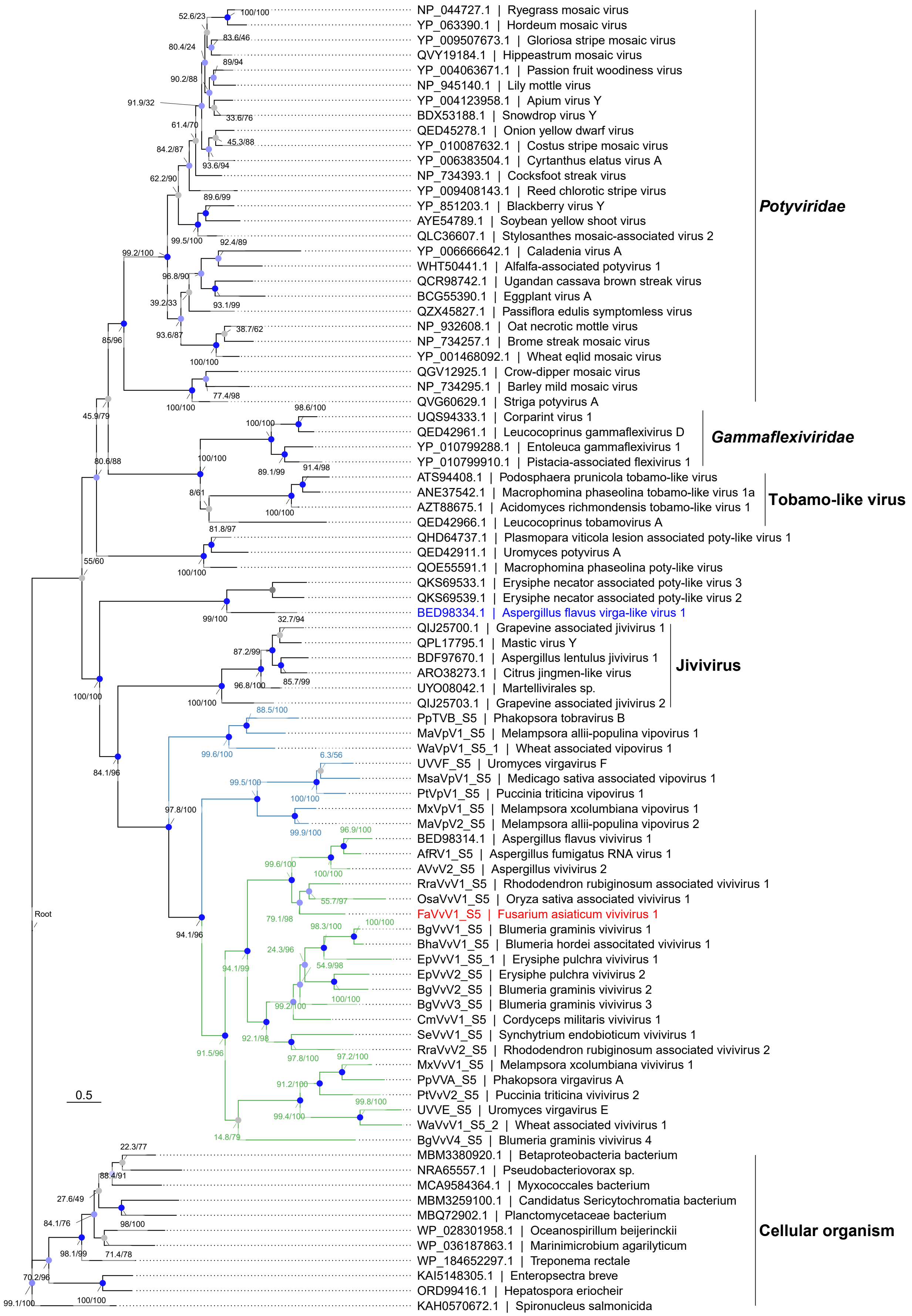

Fig. S11. Alignment of the papain-like cysteine protease domain with all gaps and unconserved region displayed, which was the full version of Fig. 6B.

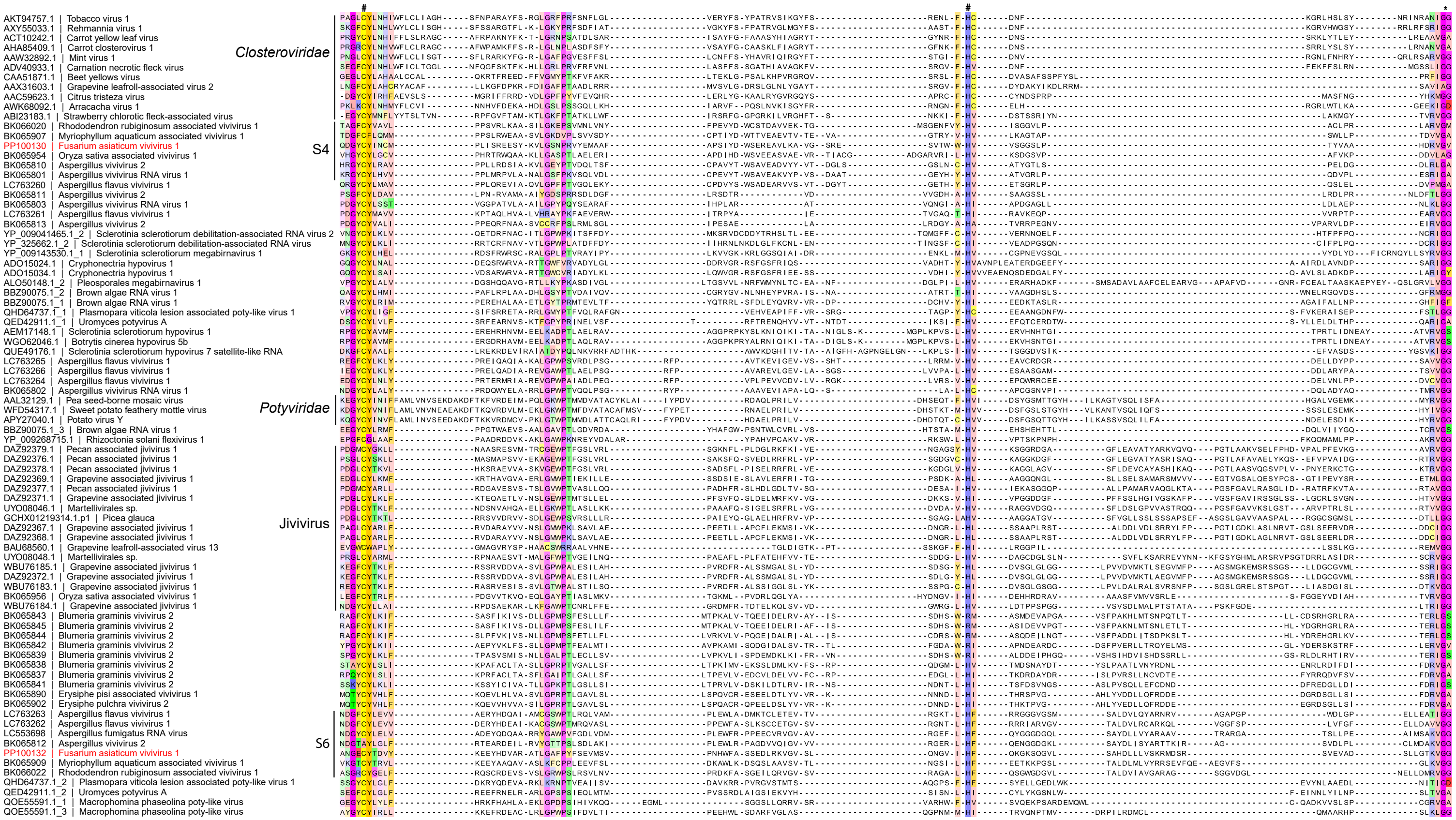

Fig. S12. (A) Schematic diagram of primers for discriminating between S9 and S10 of FaVvV1. (B) RT-PCR analysis of FaVvV1 and FaMV1 in conidial progeny of strain BZ6. (C) Verification of FaVvV1 elimination through detection in conidial progeny after a single subculture. (D) Detection of FaVvV1 in ascospore progeny. NTC, non-template control.

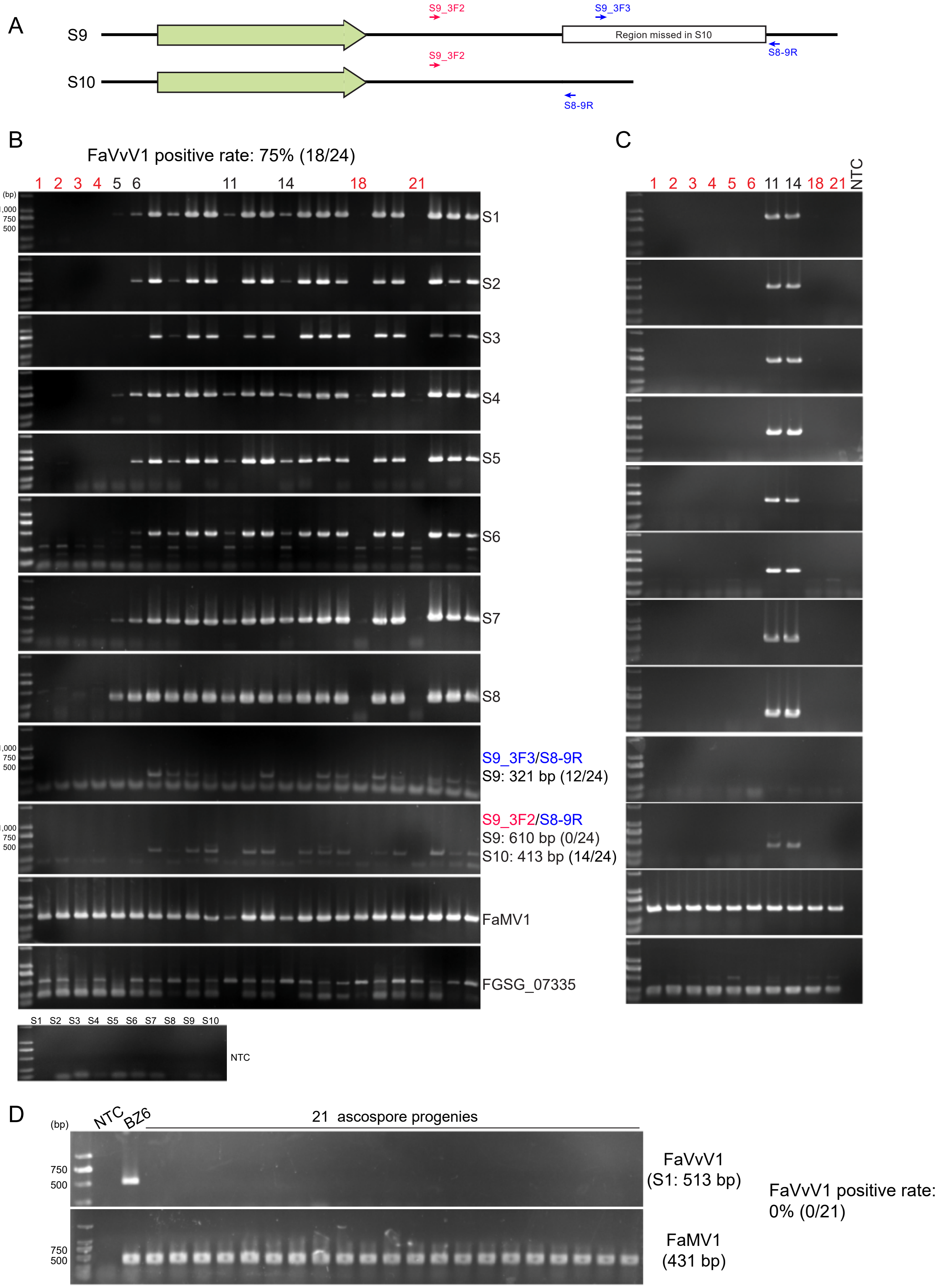

Supplement: Supplemental figures — Figures S1 to S12. [file jvi.00332-25-s0001.pdf]
