# Supplementary material for: A group of segmented viruses contains genome segments sharing homology with multiple viral taxa
Source: J Virol. 2025 Jun 4;99(7):e00332-25. doi: 10.1128/jvi.00332-25 (PMC12282112; doi:10.1128/jvi.00332-25)
Supplement: Supplemental tables — Tables S1 to S6. [file jvi.00332-25-s0002.pdf]

Table S1. Known viviruses in Genbank database. Mtr, methyltransferase. RdRP, RNA-dependant RNA polymerase. SF1H, superfamily 1 helicase. HP, hypothetical protein.

Virus name	Number of known segments	Putative host	GenBank accession	Putative functional domain	Published or submitted ([S]) by
Luckshill virus	1	*Drosophila suzukii*	MF893250	Mtr-RdRP	Medd et al., 2018
Cyril virus	1	*Drosophila suzukii*	MF893263	Mtr-SF1H	
Plasmopara viticola lesion associated vivivirus 1	**2**	*Plasmopara viticola*	MT338016	Mtr-RdRP	Chiapello et al., 2020
			MT338017	Mtr-SF1H	
Plasmopara viticola lesion associated vivivirus 2	**2**	*Plasmopara viticola*	MT338018	Mtr-RdRP	
			MT338019	Mtr-SF1H	
Plasmopara viticola lesion associated vivivirus 3	**2**	*Plasmopara viticola*	MT338020	Mtr-RdRP	
			MT338021	Mtr-SF1H	
Plasmopara viticola lesion associated vivivirus 4	**2**	*Plasmopara viticola*	MT338022	Mtr-RdRP	
			MT338023	Mtr-SF1H	
Macrophomina phaseolina tobamo-like virus 2	1	*Macrophomina phaseolina*	MT062438	Mtr-RdRP	Wang et al., 2020
Macrophomina phaseolina ilar-like virus	1	*Macrophomina phaseolina*	MT062434	Mtr-SF1H	
Phakopsora virgavirus A	1	*Phakopsora pachyrhizi*	MK231089	Mtr-RdRP	Jo et al., 2020
Phakopsora tobravirus B	1	*Phakopsora pachyrhizi*	MK231027	Mtr-SF1H	
Uromyces virgavirus E	1	*Uromyces appendiculatus*	MK231029	Mtr-RdRP	
Uromyces virgavirus F	1	*Uromyces appendiculatus*	MK231062	Mtr-RdRP	
Uromyces virgavirus C	1	*Uromyces appendiculatus*	MK231093	Mtr-SF1H	
Uromyces virgavirus G	1	*Uromyces appendiculatus*	MK231115	Mtr-SF1H	
Grapevine-associated virga-like virus 3	1	Unknown	MW648541	Mtr-RdRP	Nerva et al., 2021
Grapevine-associated virga-like virus 1	1	Unknown	MW648539	Mtr-SF1H	
Grapevine toga-like virus	1	Unknown	MT682063	Mtr-RdRP	Velasco et al., 2021
Sisal-associated virgavirus A	1	Unknown	MZ329757	Mtr-RdRP	Quintanilha-Peixoto et al,. 2021
Sisal-associated Virgavirus C	1	Unknown	MZ329763	Mtr-SF1H	
Aspergillus flavus vivivirus 1	**3**	*Aspergillus flavus*	MZ600066	Mtr-SF1H	Degola et al., 2021
			MZ600067	Mtr-RdRP	
			MZ600068	HP	
Aspergillus fumigatus RNA virus 1	**3**	*Aspergillus fumigatus*	LC553696	Mtr-SF1H	Chiba et al., 2021
			LC553697	Mtr-RdRP	
			LC553698	HP	
Wheat associated vipovirus	**2**	Unknown	MZ501227	Mtr-RdRP	Redila et al., 2022
			MZ501228	S2H	
Triticum aestivum associated virga-like virus 1	**2**	Unknown	OL519585	Mtr-RdRP	Li et al., 2022
			OL519586	Mtr-SF1H	
Plant associated virga-like virus 1	1	Unknown	OL472273	Mtr-RdRP	Rivarez et al., 2022
Plant associated virga-like virus 1	1	Unknown	OL472274	Mtr-RdRP	
Erysiphe necator associated ssRNA virus 9	1	Unknown	OL472270	Mtr-SF1H	
Erysiphe necator associated ssRNA virus 9	1	Unknown	OL472272	Mtr-SF1H	
Aspergillus flavus vivivirus 1	**12**	*Aspergillus flavus*	LC763256	Mtr-RdRP	Kuroki et al., 2023
			LC763257	Mtr-SF1H	
			LC763258	HP	
			LC763259	HP	
			LC763260	HP	
			LC763261	HP	
			LC763262	HP	
			LC763263	HP	
			LC763264	HP	
			LC763265	HP	
			LC763266	HP	
			LC763267	HP	
Botryosphaeria dothidea tobamo-like virus	1	*Botryosphaeria dothidea*	MK189194	Mtr-RdRP	Bian et al., 2019[S]
Botryosphaeria dothidea bromo-like virus 1	1	*Botryosphaeria dothidea*	MT103583	Mtr-SF1H	Yang et al., 2020[S]
Leveillula taurica associated virga-like virus 1	1	*Leveillula taurica*	MN609856	Mtr-SF1H	Chiapello et al., 2020[S]
Erysiphe necator associated virga-like virus 3	1	*Erysiphe necator*	MN627437	Mtr-RdRP	Rodriguez-Romero et al., 2021[S]
Erysiphe necator associated virga-like virus 4	1	*Erysiphe necator*	MN627438	Mtr-RdRP	
Erysiphe necator associated virga-like virus 5	1	*Erysiphe necator*	MN627449	Mtr-RdRP	
Erysiphe necator associated virga-like virus 6	1	*Erysiphe necator*	MN627455	Mtr-RdRP	
Erysiphe necator associated virga-like virus 7	1	*Erysiphe necator*	MN627460	Mtr-RdRP	
Erysiphe necator associated virga-like virus 8	1	*Erysiphe necator*	MN627467	Mtr-RdRP	
Erysiphe necator associated virga-like virus 9	1	*Erysiphe necator*	MN627477	Mtr-RdRP	
Erysiphe necator associated virga-like virus 11	1	*Erysiphe necator*	MN627476	Mtr-RdRP	
Erysiphe necator associated virga-like virus 12	1	*Erysiphe necator*	MN627478	Mtr-RdRP	
Erysiphe necator associated virga-like virus 13	1	*Erysiphe necator*	MN627446	Mtr-RdRP	
Erysiphe necator associated ssRNA virus 2	1	*Erysiphe necator*	MN558699	Mtr-SF1H	
Erysiphe necator associated ssRNA virus 4	1	*Erysiphe necator*	MN627435	Mtr-SF1H	
Erysiphe necator associated ssRNA virus 5	1	*Erysiphe necator*	MN627436	Mtr-SF1H	
Erysiphe necator associated ssRNA virus 6	1	*Erysiphe necator*	MN627445	Mtr-SF1H	
Erysiphe necator associated ssRNA virus 7	1	*Erysiphe necator*	MN627451	Mtr-SF1H	
Erysiphe necator associated ssRNA virus 8	1	*Erysiphe necator*	MN627454	Mtr-SF1H	
Erysiphe necator associated ssRNA virus 9	1	*Erysiphe necator*	MN627473	Mtr-SF1H	
Erysiphe necator associated ssRNA virus 10	1	*Erysiphe necator*	MN627475	Mtr-SF1H	
Erysiphe necator associated ssRNA virus 11	1	*Erysiphe necator*	MN627484	Mtr-SF1H	
Erysiphe necator associated ssRNA virus 12	1	*Erysiphe necator*	MN627488	Mtr-SF1H	
Erysiphe necator associated ssRNA virus 13	1	*Erysiphe necator*	MN627450	Mtr-SF1H	
Hangzhou virga-like virus	1	Unknown	OM514354	Mtr-RdRP	Feng et al., 2022[S]
Botryosphaeria dothidea vivivirus 1	**2**	*Botryosphaeria dothidea*	MW872419	Mtr-SF1H	Liu et al., 2022[S]
			MW872420	Mtr-RdRP	

Table S2. Results of searching the uniprot_sprot_vir70 and pdb30 database with HHpred (Cutoff: Probability > 90%).

Query	ID (UniProtKB / PDB)	Hit.Description	Hit.Taxonomy	Q.Start	Q.End	Q.Length	T.Start	T.End	T.Length	Aligned_co	E.value	Identities	Probab.	Score	Similarity	Template_Neff
FaVvV1_S8	Q08531	Capsid protein of Beet yellows virus (isolate Ukraine)	*Closteroviridae, Closterovirus*	80	268	295	15	204	204	166	0.007	17	96.95	52.61	0.14	7.6
FaVvV1_S8	O41518	Capsid protein of Grapevine leafroll-associated virus 3 (isolate United States/NY1)	*Closteroviridae, Ampelovirus*	80	258	295	125	305	313	159	0.064	10	96.65	50.68	-0.05	6.2
FaVvV1_S8	Q83049	Capsid protein of Lettuce infectious yellows virus (isolate United States/92)	*Closteroviridae, Crinivirus*	77	249	295	57	230	249	153	0.054	11	96.32	49.37	0.073	6.6
FaVvV1_S8	Q08538	Minor capsid protein of Beet yellows virus (isolate Ukraine)	*Closteroviridae, Closterovirus*	80	249	295	32	198	216	151	0.042	13	96.24	48.3	0.111	7.5
FaVvV1_S8	O41519	Minor capsid protein of Grapevine leafroll-associated virus 3 (isolate United States/NY1	*Closteroviridae, Ampelovirus*	81	250	295	294	463	477	150	0.11	17	95.77	52.3	0.064	4.8
FaVvV1_S8	Q00686	Capsid protein of Citrus tristeza virus (isolate T36)	*Closteroviridae, Closterovirus*	80	250	295	39	206	223	150	0.1	20	95.52	46.27	0.019	7.3
FaVvV1_S8	P83574	Coat protein of Nigerian sorghum potyvirus	*Potyviridae, Potyvirus*	85	250	295	108	257	293	140	0.67	13	93.5	44.17	0.128	5
FaVvV1_S8	8ACC_A	Coat protein of Sweet potato feathery mottle virus	*Potyviridae, Potyvirus*	85	250	295	107	265	302	148	0.61	14	92.59	44.4	0.136	5.4
FaVvV1_S8	Q65729	Coat protein of Brome streak virus (strain German)	*Potyviridae, Tritimovirus*	85	250	295	731	889	942	140	1.5	14	91.58	45.65	0.135	8.5
FaVvV1_S8	P32575	Coat protein of Passionfruit woodiness virus (strain Severe)	*Potyviridae, Potyvirus*	85	250	295	82	231	269	140	0.94	14	91.31	42.52	0.117	5.4
FaVvV1_S8	P20177	Coat protein of Clover yellow vein virus	*Potyviridae, Potyvirus*	85	250	295	206	355	391	140	1.2	14	90.46	42.69	0.14	7.3
FaVvV1_S8	6HXX_BB	Coat protein of Potato virus Y	*Potyviridae, Potyvirus*	85	250	295	81	230	267	140	1.9	13	90.35	40.32	0.144	5.7

Table S3. Results of searching the available protein structure database with the predicted FaVV1 CP 3D structure using Foldseek in 3Di/AA mode (Cutoff: Probability > 40%).

Query	Database	Target	Description	Scientific Name	Prob.	Seq. Id.	E-Value	Score	Query Pos.	Target Pos.
FaVvV1_S8	PDB100	8acc_A	CryoEM structure of sweet potato mild mottle virus VLP	Sweet potato mild mottle virus	1	13.2	0.00383	119	45-272 (294)	4-223 (231)
FaVvV1_S8	PDB100	8acb_A	CryoEM structure of sweet potato feathery mottle virus VLP	Sweet potato feathery mottle virus	0.99	13.5	0.0699	81	64-281 (294)	15-207 (220)
FaVvV1_S8	PDB100	6hxx_AA	Potato virus Y	Potato virus Y	0.96	10	0.13	74	67-273 (294)	21-201 (224)
FaVvV1_S8	PDB100	6hxz_a	Virus-like Particles based on Potato Virus Y	Potato virus Y	0.41	8	2.26	48	68-235 (294)	23-174 (176)
FaVvV1_S8	AFDB50	AF-Q5ZF65-F1-model_v4	RdRP_1 domain-containing protein (Note: unknown potyvirus infected)	Plantago major	1	13.2	0.0437	105	1-274 (294)	176-424 (441)
FaVvV1_S8	AFDB50	AF-A0A4T9WAG3-F1-model_v4	POTEX_CARLAVIRUS_COAT domain-containing protein	Tissierella creatinini	1	14	0.032	93	1-260 (294)	34-304 (325)
FaVvV1_S8	AFDB50	AF-A0A1W5RMM0-F1-model_v4	Uncharacterized protein	Pediastrum duplex	0.99	10.2	0.0628	88	1-246 (294)	9-255 (270)

Table S4. The genome segments and accession number of vivivirids mentioned in this study. (Putative paralogous segments of the same virus were highlight in blue. N.D., not detected)

Virus name	Virus abbr.	S1 (VP1: Mtr1-RdRp)	S2 (VP2: Mtr2-SF1H)	S3 (VP3: chymotrypsin-type serine protease)	S4 (VP4: papain-like cysteine protease)	S5 (VP5: SF2 helicase)	S6 (VP6: papain-like cysteine protease)	S7 (VP7)	S8 (VP8: capsid)	S9 & S10	papain-like cysteine protease	papain-like cysteine protease	SRR accession	Segment No.	Sequence reference
Blumeria hordei associtated vivivirus 1	BhaVvV1	BK065861	BK065862	BK065863	N.D.	BK065864	N.D.	N.D.	BK065865 BK065866	N.D.	N.D.	N.D.	SRR3657679	6	This study
Blumeria graminis vivivirus 1	BgVvV1	BK065827	BK065828	BK065829	N.D.	BK065830	N.D.	N.D.	BK065831 BK065832	N.D.	N.D.	N.D.	SRR14129631	6	This study
Erysiphe pulchra vivivirus 1	EpVvV1	BK065892	BK065893	BK065894	N.D.	BK065895 BK065896	N.D.	N.D.	BK065897	N.D.	N.D.	N.D.	SRR10619097	6	This study
Erysiphe pisi associated vivivirus 1	EpaVvV1	BK065886	BK065887	BK065888	N.D.	BK065889	N.D.	N.D.	BK065891	N.D.	N.D.	BK065890	SRR7066906	6	This study
Erysiphe pulchra vivivirus 2	EpVvV2	BK065898	BK065899	BK065900	N.D.	BK065901	N.D.	N.D.	BK065903	N.D.	N.D.	BK065902	SRR10619097	6	This study
Blumeria graminis vivivirus 2	BgVvV2	BK065833	BK065834	BK065835	N.D.	BK065836	N.D.	N.D.	BK065847 BK065848	N.D.	N.D.	BK065837 BK065838 BK065839 BK065840 BK065841 BK065842 BK065843 BK065844 BK065845	SRR14129631	15	This study
Blumeria graminis vivivirus 3	BgVvV3	BK065849 BK065850	BK065851	BK065852	N.D.	BK065853	N.D.	N.D.	BK065854 BK065855	N.D.	N.D.	N.D.	SRR14129631	7	This study
Cordyceps militaris vivivirus 1	CmVvV1	BK065867	BK065868	BK065869	N.D.	BK065870	N.D.	N.D.	BK065872	N.D.	BK065871		SRR8640098	6	This study
Synchytrium endobioticum vivivirus 1	SeVvV1	BK066030	BK066031	BK066032	N.D.	BK066033	N.D.	N.D.	BK066036	N.D.	BK066034	BK066035	SRR8074753	7	This study
Rhododendron rubiginosum associated vivivirus 2	RraVvV2	BK066024	BK066025	BK066026	N.D.	BK066027	N.D.	N.D.	BK066029	N.D.	BK066028	N.D.	SRR9128705	6	This study
Phakopsora virgavirus A	PpVVA	BK065979	BK065980	BK065981	N.D.	BK065982	N.D.	N.D.	BK065983	N.D.	N.D.	N.D.	SRR609193 SRR616699 SRR61700 SRR8181606	5	Jo et al., 2022; this study
Melampsora xcolumbiana vivivirus 1	MxVvV1	BK065942	BK065943	BK065944 BK065946 BK065947	N.D.	BK065945	N.D.	N.D.	BK065948 BK065949 BK065950	N.D.	N.D.	N.D.	SRR10229823	9	This study
Puccinia triticina vivivirus 2	PtVvV2	BK066012	BK066013	BK066014	N.D.	BK066015	N.D.	N.D.	BK066016	N.D.	N.D.	N.D.	SRR14129631 SRR5810395	5	This study
Uromyces virgavirus E	UVVE	BK066047	BK066048	BK066049	N.D.	BK066050	N.D.	N.D.	BK066051	N.D.	N.D.	N.D.	SRR609082	5	Jo et al., 2022; this study
Puccinia triticina vivivirus 1	PtVvV1	BK066006	BK066007	BK066008	N.D.	BK066009 BK066010	N.D.	N.D.	BK066011	N.D.	N.D.	N.D.	SRR14239692 SRR5810395 SRR14129631	6	This study
Blumeria graminis vivivirus 4	BgVvV4	BK065856	BK065857	BK065859	N.D.	BK065858	N.D.	N.D.	BK065860	N.D.	N.D.	N.D.	SRR14129631	5	This study
Rhododendron rubiginosum associated vivivirus 1	RraVvV1	BK066017	BK066018	BK066019	BK066020	BK066021	BK066022	N.D.	BK066023	N.D.	N.D.	N.D.	SRR9128705	7	This study
Fusarium asiaticum vivivirus 1	FaVvV1	PP100127	PP100128	PP100129	PP100130	PP100131	PP100132	PP100133	PP100134	PP100135 PP100136	N.D.	N.D.	SRR27452104	10	This study
Myriophyllum aquaticum associated vivivirus 1	MaaVvV1	BK065904	BK065905	BK065906	BK065907	BK065908	BK065909	N.D.	BK065910	N.D.	N.D.	N.D.	SRR8100641	7	This study
Synchytrium endobioticum vivivirus 1	OsaVvV1	BK065951	BK065952	BK065953	BK065954	BK065955	BK065956	N.D.	BK065958	N.D.	BK065957	N.D.	SRR11243206	8	This study
Aspergillus vivivirus 2	AVvV2	BK065806	BK065807	BK065808	BK065810	BK065809	BK065812	N.D.	BK065814	N.D.	BK065811 BK065813	N.D.	SRR15319217	9	This study
Aspergillus fumigatus RNA virus 1	AfRV1	LC553696	LC553697	BK065799	BK065801	BK065800	LC553698	N.D.	BK065805	N.D.	BK065803 BK065804	BK065802	DRR235409	10	Chiba et al. 2021; Degola et al., 2021; this study
Aspergillus flavus vivivirus 1	AfVvV1	LC763256	LC763257	LC763258	LC763260	LC763259	LC763262 LC763263	N.D.	LC763267	N.D.	LC763261	LC763264 LC763265 LC763266	DRR452452	12	Kuroki et al. 2021
Uromyces virgavirus F	UVVF	BK066052	BK066053	BK066054	N.D.	BK066055	N.D.	N.D.	BK066056	N.D.	N.D.	N.D.	SRR609082	5	Jo et al., 2022; this study
Puccinia triticina vipovirus 1	PtVpV1	BK066000	BK066001	BK066002	N.D.	BK066003	N.D.	N.D.	BK066004 BK066005	N.D.	N.D.	N.D.	SRR14129631 SRR5810395	6	This study
Medicago sativa associated vipovirus 1	MsaVpV1	BK065931	BK065932	BK065933	N.D.	BK065934	N.D.	N.D.	BK065935	N.D.	N.D.	N.D.	SRR7193614	5	This study
Melampsora xcolumbiana vipovirus 1	MxVpV1	BK065936	BK065937	BK065938	N.D.	BK065939	N.D.	*N.D.*	BK065940 BK065941	N.D.	N.D.	N.D.	SRR10229823	6	This study
Melampsora allii-populina vipovirus 2	MaVpV2	BK065925	BK065926	BK065927	N.D.	BK065928	N.D.	N.D.	BK065929 BK065930	N.D.	N.D.	N.D.	SRR4063167	6	This study
Wheat associated vipovirus 1	WaVpV1	MZ501227	BK066057	BK066058	N.D.	MZ501228 BK066059	N.D.	N.D.	BK066060	N.D.	N.D.	N.D.	SRR14239692 SRR14129631 SRR5810395	6	Redila et al., 2021; this study
Phakopsora tobravirus B	PpTVB	BK065974	BK065975	BK065976	N.D.	BK065977	N.D.	N.D.	BK065978	N.D.	N.D.	N.D.	SRR609193 SRR616699 SRR61700 SRR8181606	5	Jo et al., 2022; this study
Melampsora allii-populina vipovirus 1	MaVpV1	BK065920	BK065921	BK065922	N.D.	BK065923	N.D.	N.D.	BK065924	N.D.	N.D.	N.D.	SRR4063167	5	This study

Table S5. The genome segments and accession number of pucciniviruses mentioned in this study. (Homolougs segments of the same virus were highlight in blue. N.D., not detected; "small" refers to unknown segments with small peptides (< 200 aa); "UN" means unknown segments).

Virus name	Virus abbr.	Mtr1-RdRp	Mtr2-SF1H	Mtr	RNase III	S5 (unknown helicase)	UN	capsid	small1	small2	small3	small4	UN1	UN2	UN3	SRR accession	Segment No.	Sequence reference
Puccinia triticina puccinivirus 1	PtPcV1	BK065985	BK065984	BK065990	BK065995	BK065989	BK065992 BK065993 BK065994	BK065996	BK065998	BK065997	BK065999	N.D.	BK065986 BK065987 BK065988	BK065991	N.D.	SRR5810395 SRR14129631	16	This study
Puccinia coronata puccinivirus 1	PcPcV1	BK065960	BK065959	BK065964	BK065968	BK065963	BK065966 BK065967	BK065969	BK065971	BK065970	BK065972	BK065973	BK065961 BK065962	BK065965	N.D.	SRR5976052	15	This study
Uromyces virus A	UVA	BK066038	BK066037	BK066040	BK066043	BK066039	BK066042	BK066044	BK066046	BK066045	N.D.	N.D.	N.D.	BK066041	N.D.	SRR609082	10	Jo et al., 2022; this study
Melampsora allii-populina puccinivirus 1	MaPcV1	BK065912	BK065911	BK065914	BK065916	N.D.	BK065915	BK065917	BK065919	BK065918	N.D.	N.D.	BK065913	N.D.	N.D.	SRR4063167	9	This study
Erysiphe pisi associated puccinivirus 1	EpaPcV1	BK065874	BK065873	N.D.	BK065879 BK065880	BK065875 BK065876 BK065877 BK065878	N.D.	BK065885	N.D.	N.D.	N.D.	N.D.	N.D.	N.D.	BK065881 BK065882 BK065883 BK065884	SRR7066906	13	This study
Blumeria graminis puccinivirus 1	BgPcV1	BK065816	BK065815	N.D.	BK065819 BK065821	BK065817 BK065818	N.D.	BK065822 BK065825	N.D.	N.D.	N.D.	N.D.	N.D.	N.D.	BK065820 BK065823 BK065824 BK065826	SRR14129631	12	This study

Table S6. Primers used in this study.

Primer name	Sequence	Application and PCR product length
Fg16F	CTCCGGATATGTTGCGTCAA	molecular identification of *Fusarium* spp.
Fg16R	GGTAGGTATCCGACATGGCAA	molecular identification of *Fusarium* spp.
ITS1	TCCGTAGGTGAACCTGCGG	molecular identification of *Fusarium* spp.
ITS4	TCCTCCGCTTATTGATATGC	molecular identification of *Fusarium* spp.
EF1T	ATGGGTAAGGAGGAC AAGAC	molecular identification of *Fusarium* spp.
EF2T	GGAAGTACCAGTGATCA TGTT	molecular identification of *Fusarium* spp.
S1F	AGAAAGCGCGTGTCTGAGAA	824 bp
S1R	CTACCCTCGATACGCACCAC	824 bp
S2F	AAACAACGTGCGCTCAACTC	724 bp
S2R	CACGCAGTATGCGAAACGAG	724 bp
S3F	ACGTGAAGGTAAGCAAGCGA	713 bp
S3R	TGTCTTCCCTCGCAAACTCC	713 bp
S4F	GGCAAAGCAGGATGGGTACT	647 bp
S4R	TCCCACGCGACATTGAAAGA	647 bp
S5F	AACGCGGTTTTCAACTCTGC	611 bp
S5R	CGACGACACACAGACACTCA	611 bp
S6F	TCGGATGATTGGCTCGATGG	506 bp
S6R	TTCTGAAGCGAGACACCCAC	506 bp
S7F	GAAGGTCTTCGTGAGGCCAA	439 bp
S7R	TGGCACAAGAAACCAAGGGT	439 bp
S8F	ACAACAGTGGCAAGTCTGCT	335 bp
S8R	AGGCTGCTGAAGAGACGAAC	335 bp
S8R2	GCCACGTAACAACAACCACA	845 bp, comfirm the sequence of S8 with primer S8F
S9F	TGTCGATTGGGGTCTGTGTG	366 bp
S9R	ACCAGGCATCAACATACGCA	366 bp
S8-9R	CGAAAGCCCAGTGCAATTAC	S8F (1078 bp), with S9_3F2 (two bands: 610 bp for S9, 413 bp for S10)
RNA1F	CCGATGCAAAACCGCCTATG	513 bp, detection S1 with primer RNA1F
RNA1R	CCCAAATCACCCTGGTACCC	513 bp, detection S1 with primer RNA1F (vertical transmission assay)
RNA2F	AGACCGTTGCACACCATGAT	911 bp, detection S2 with primer RNA2R
RNA2R	GGTCTCATGGCAAAGGCTCT	911 bp, detection S2 with primer RNA2F (vertical transmission assay)
RNA6F	CGTCTACTGTCGGTATGGGC	590 bp, detection S6 with primer RNA6R, 2 SNP
RNA6R	GTTCTGAAGCGAGACACCCA	590 bp, detection S6 with primer RNA6F (vertical transmission assay)
FaMV1F	TCAGTGGCTGCGCTAAGATC	431 bp, detection FaMV1 with primer FaMV1R, 2 SNP
FaMV1R	GGTAACCAAATCGGTGCTGCTTTC	431 bp, detection FaMV1 with primer FaMV1F (vertical transmission assay)
b-Tubulin_F	ACCCTCAGTGTAGTGACCCTTGGC	286 bp (gDNA), 244 bp (cDNA), beta-tubulin
b-Tubulin_R	AAAGCTGGTCCGAACTCTCA	286 bp (gDNA), 244 bp (cDNA), beta-tubulin
PC3-T7 loop	GGATCCCGGGAATTCGGTAATACGACTCACTATATTTTTATAGTGAGTCGTATTA	phosphorated at the 5' end
PC2	CCGAATTCCCGGGATCC	
RT-adaptor +A tail	GAGGACTCGAGCTCAAGCATGCATTTTTTTTTTTTTTTT	5/3' RACE
PCR primer 1 - tail adaptor	GAGGACTCGAGCTCAAGC	5/3' RACE
RT-adaptor+G	GAGGACTCGAGCTCAAGCATGCATCCCCCCCCCCCCCCC	5/3' RACE
S1_5R1	ATCGGCAACAGCTATCACTT	459 bp, 5' RACE
S1_5R2	GGACACAAGTCAAGCTGTTC	367 bp, 5' RACE
S1_3F1	TGGGATGACAAGTTCGAGAG	338 bp, 3' RACE
S1_3F2	CTTAACGGTTTGGCAGAAGG	278 bp, 3' RACE
S2_5R1	GATGAGAAATGTAGCGTGCC	872 bp, 5' RACE
S2_5R2	CATCCTTCGGATCGTGTAGT	613 bp, 5' RACE
S2_3F1	ACACCATTTTCAGATTCGGC	605 bp, 3' RACE
S2_3F2	GATCTTGGCCTGTGTCAATC	580 bp, 3' RACE
S3_5R1	TACCTTCACGTTTGGCCTTA	490 bp, 5' RACE
S3_5R2	TAAGTCTGTCTGCGTGTACC	472 bp, 5' RACE
S3_3F1	GTGGCGTTCCTAAATTCGAG	724 bp, 3' RACE
S3_3F2	GAAGGTGTCTCACACACAGA	639 bp, 3' RACE
S4_5R1	CACCTGATCTCGGCTCTATG	537 bp, 5' RACE
S4_5R2	CATCAGACACGCAAGAACAG	469 bp, 5' RACE
S4_3F1	TGGTGACATGAAGATACGCA	675 bp, 3' RACE
S4_3F2	CGAGCTTCAAGGAGTTGTTG	384 bp, 3' RACE
S5_5R1	TCAACACCTTTCGACTTCCA	522 bp, 5' RACE
S5_5R2	TGCAAGCAACCTAGAAGGAA	370 bp, 5' RACE
S5_3F1	AATCGGTGTATGCAGAGGAG	431 bp, 3' RACE
S5_3F2	TGTGATGAGCATCTGGTTCA	403 bp, 3' RACE
S6_5R1	GGAACCGACATGACTTCAGA	607 bp, 5' RACE
S6_5R2	CAACATCCCTGCCAGAGTTA	504 bp, 5' RACE
S6_3F1	ACTACAGGTACGCTTTGGAG	563 bp, 3' RACE
S6_3F2	TGTCTTTGGGAATTGCGTTC	445 bp, 3' RACE
S7_5R1	AACGCGAACACACGATTAAG	517 bp, 5' RACE
S7_5R2	ATGTTTGACCTCGCCAAAAG	467 bp, 5' RACE
S7_3F1	AGGGGTTCCTTTTGAACCTT	444 bp, 3' RACE
S7_3F2	CTTGCGTGATAAAGCTCCTG	378 bp, 3' RACE
S8_5R1	ACGAAAGAATCGCCTCTGAT	618 bp, 5' RACE
S8_5R2	CGCCTCCAAAAATGGAAACT	480 bp, 5' RACE
S8_3F1	GGTGGACAATCCAGTCATCT	495 bp, 3' RACE
S8_3F2	GGTGGTAGAGTTGAGGGAAG	461 bp, 3' RACE
S9_5R1	CTGCTTCTCCAAATCACTGC	410 bp, 5' RACE
S9_5R2	AAGCCAAACCATCAGAGTCA	344 bp, 5' RACE
S9_3F1	TGATTTGGAGAAGCAGGTCA	869 bp, 3' RACE
S9_3F2	TGTCACATGCGAAACACAAG	709 bp, 3' RACE, for S10 & 518 bp for S9
S9_3F3	GACTTTGTGTTGTGGCGGTT	420 bp, 3' RACE, specific for S9, not S10; amplify 321 bp with S8-9R
